# Supplementary material for: Direct serotonin release in humans shapes aversive learning and inhibition
Source: Nat Commun. 2024 Aug 9;15:6617. doi: 10.1038/s41467-024-50394-x (PMC11315928; doi:10.1038/s41467-024-50394-x)
Supplement: Supplementary file 1 — Supplementary Information [file 41467_2024_50394_MOESM1_ESM.pdf]

Supplementary Information

Title: Direct serotonin release in humans shapes aversive learning and inhibition

Authors: Michael J Colwell [1,2*], Hosana Tagomori [1,2], Fei Shang [1,2], Hoi Iao Cheng [1,2], Chloe Wigg [1,2], Michael Browning [1,2], Philip J Cowen[1,2], Susannah E Murphy [1,2,a], & Catherine J Harmer [1,2 a*]

[1] *University Department of Psychiatry, University of Oxford, Warneford Hospital, Oxford, UK*

[2] *Oxford Health NHS Foundation Trust, Warneford Hospital, Oxford, UK;*

[a] *These authors jointly supervised this work.*

***Corresponding authors:**

Michael J Colwell; Email: michael.colwell@psych.ox.ac.uk; Tel: +44 (0)1865 618200;

Catherine J Harmer; Email: catherine.harmer@psych.ox.ac.uk; Tel: +44 (0)1865 618326

Supplementary Methods

Eligibility Criteria

Inclusion criteria:

- Participant is willing and able to give informed consent for participation in the research
- Not currently taking any medications (except the contraceptive pill)
- Aged 18 – 22 years
- Male or female
- Sufficiently fluent English to understand and complete the task
- Body Mass Index above 18-30
- Weight of 40-75kg

Exclusion criteria:

- Current pregnancy (as determined by urine pregnancy test taken during Screening and First Dose Visit) or breast feeding
- Any past or current Axis 1 DSM-V psychiatric disorder
- Clinically significant abnormal values for liver function tests, clinical chemistry, urine drug screen, blood pressure measurement and ECG. A participant with a clinical abnormality or parameters outside the reference range for the population being studied may be included only if the Investigator considers that the finding is unlikely to introduce additional risk factors and will not interfere with the study procedures
- History of, or current medical conditions which, in the opinion of the investigator, may interfere with the safety of the participant or the scientific integrity of the study, including epilepsy/seizures, brain injury, hepatic or renal disease, severe gastro-intestinal problems, Central Nervous System (CNS) tumours, neurological conditions
- Current or past history of drug or alcohol dependency
- Use of recreational drugs (e.g. cannabis, cocaine, amphetamines) within past 3 months
- Participation in a study which uses the same computer tasks as those in the present study (determined by asking participants about previous studies participated in during screening)
- Participation in a study that involves the use of a medication within the last three months
- Smoking > 5 cigarettes per day
- Typically drinks > 6 caffeinated drinks per day (e.g. tea, coffee, coca cola, Red Bull)
- Participant is unlikely to comply with the clinical study protocol or is unsuitable for any other reason, in the opinion of the Investigator
- Current or recent use (≤1 year) of 3,4-Methylenedioxymethamphetamine (MDMA)

Saliva sample collection and processing

To determine cortisol levels, saliva samples were collected from participants immediately before taking the initial dose of the drug or placebo (baseline), one hour post-dose, and three hours post-dose. Saliva samples were collected via Salivette® Cortisol synthetic swabs. Within a 2-3 hours of collecting samples, the samples were transported to a laboratory where they were rendered acellular by centrifugation upon receipt. Prior to immunoassay, all samples were stored in the Neurosciences Building (Department of Psychiatry) in a de-identified form at -30°C. When the randomisation code was broken, all samples were immunoassayed over linear calibration curves via a Salimetrics salivary cortisol ELISA kit (#1-3002) according to the manufacturer protocol [1]. Cortisol concentration is expressed as a measure of μg/dL. Salivary cortisol was analysed across three

timepoints (before dose, 1-hr and 3-hr post-dose) using mixed linear effects modelling using time-by-allocation as an interaction term.

Cognitive and Emotional Task Battery

The Probabilistic Instrumental Learning task (Version 220916, adapted from [2]) measures reward and loss sensitivity following instrumental learning (Fig. 2A, main paper). This task requires participants to select between one of a pair of symbols per trial; two novel pairs of symbols alternate throughout task blocks, with one pair representing a high-probability-win condition (outcomes: 20p gain or no change) or a high-probability-loss condition (outcomes: 20p loss or no change). For each pair, symbols are tied to reciprocal probability values of 70% or 30%, where the outcome of selection is displayed following each trial. Participants were instructed to select outcomes most likely to translate to maximal monetary gain which would are awarded to them at study completion. Variables which task records includes response time, optimal selection per trial paradigm, and total gained. Per previous implementations of the task, only the last 40 trials per block (20 high-probability loss, 20 high-probability win) were included in the non-model analysis of optimal choice behaviour, as this is where the learning plateau typically occurs [2,3] . Using non-model task data, learning curves were generated as a temporal-learning representation of optimal selection across each trial paradigm per block (Fig. 1B, main paper).

The Affective Go/No-Go Task [AGNG] (Version 1.2) [4] measures executive control and impulsivity in under conditions of affective interference (Fig. 3A, main paper). During the affective interference condition, blue and yellow rectangles are superimposed on emotional distractor images which were pairs of eyes expressing ‘fear’ or ‘happy’ emotions. The non-affective control condition displays blue and yellow rectangles superimposed on scrambled versions of the emotional distractor images with matched cropping and luminance. The task is balanced across three block types depending on the condition during the inhibitory stimuli (*i.e.,* ‘fearful distractor’, ‘happy distractor’ and ‘no distractor/scramble’). Before each block new rules are given for participants (*e.g.,* “If you see a blue image, do not press spacebar”). Inhibitory (‘no-go’) and input required (‘go’) stimuli are displayed at a 1:3 ratio, respectively. Stimuli are displayed for 400ms with interstimulus intervals displayed at a pseudorandom jitter between 1000-2000ms. The task was designed for and displayed on a high refresh rate monitor (1080p, 120hz) for greater frame time accuracy/synchronisation.

The *N*-back task is a task of verbal working memory (Version 1.2) [5], wherein a sequence of letters is displayed centre-screen for an interval of 1000ms per letter. Participants are prompted before each sequence to indicate if a letter corresponded to a previously presented letter that occurred n-trials ago (one trial ago [1-back], two trials ago [2-back], three trials ago [3-back], or if the trial contained the letter ‘x’ [0-back]). Errors of commission and omission are recorded alongside response time.

The Contextual Cueing Task (CCT; adapted from [6]) measures implicit learning and visual search ability. During the task participants are to identify the orientation of a target stimulus which was onscreen among flanker stimuli. Half of trials include a contextual cueing element where the stimulus array was identical as the subsequent trial, while the other half contains novel arrays. In line with previous analyses of the task [6], accuracy difference scores (cued – novel trials) were analysed, in addition to response time differences (cued – novel trials). These variables were analysed via mixed ANCOVA modelling with the task stage (first half [blocks 1 & 2] or second half [blocks 3 & 4]) serving as a within-subjects factor.

The Rey Auditory Verbal Learning Task (RAVLT) [7] measures episodic memory encoding, recall and retrieval. In this implementation of the task, participants are played an audio recording of 15 commonplace nouns (List A) and prompted to recall them once the recording ended. This process is

repeated five times before a novel list of commonplace nouns (List B) was played and immediate recall of the novel list was prompted. Participants are then asked to free recall List A items (short delay), and then repeat this process after a twenty minute period (long delay). Throughout the task, the number of items correctly recalled, the number of repeated recalls (repetitions) and the number of incorrect items (intrusions) are measured.

The short-form Oxford Memory Test (OMT; adapted from [8]) measures visuospatial complex working memory. During the task, 1 or 3 fractals are displayed on the screen for a brief period of 1 or 3 seconds, followed by a 1 or 4 second interstimulus period. Following this, participants are presented with two stimuli (one presented in the subsequent array and one flanker), where participants are to recall the previously presented stimuli and then drag it to its location where it was onscreen prior to the interstimulus period. Accuracy and response time for identification and localisation are collected for this task.

Participants completed all tasks in the task battery at the initial dose and follow-up visits; participants tasks in the same order each visit: 1) AVLT, 2) AGNG, 3) N-Back, 4) PILT, 5) OMT, and 6) OMT. In the randomisation algorithm, one of the two strata (alongside gender) was task stimulus version. Task stimulus version refers to versions of tasks across the cognitive and emotional task battery in which the stimuli presentation was varied to deter practice effects. There were two task stimulus versions (1 and 2) which, depending on randomisation, would be administered at the initial dose visit or follow-up visit, accordingly. All tasks within the battery were designed to have two stimulus versions except the Oxford Memory Task and Contextual Cueing Task.

Effects Size Calculations

Effect sizes were calculated for both mixed-effects ANCOVA models and EMM models. For the ANCOVA models, partial eta squared (η_p^2) was calculated by applying *eta_squared* function (from the *effectsize* R package) to the mixed effect model. For EMM comparisons, cohen's *d* was calculated by applying the *eff_size* function (from the 'emmeans' R package) to an EMM derived from the outcome variable at follow-up.

Computational Modelling – Probabilistic Instrumental Learning Task

Data from the probabilistic instrumental learning task were fit to computational reinforcement learning models implemented previously [9,10], based on the Q model described by Pessiglione *et al.* [2]. For the outcome sensitivity model, the value of selecting a stimulus was updated on a trial-by-trial basis in the model using the equation below, where the value of the non-selected stimulus was reciprocally updated:

$$Q_{t+1(s)} = Q_{t(s)} + \alpha_j \left(\rho_j R_t \; - Q_{t(s')} \right)$$

The learning expectation $Q_{t(s)}$ refers to the value attached to a stimulus/symbol (s) at a given time/trial (t), while R_t refers to the positive (coded as '1' for win in win trials, and '0' for no change in loss trials) or negative (coded as '0' for no change in win trials, and '-1' for loss in loss trials) outcome observed. Learning rate α_j and outcome sensitivity ρ_j parameters are set for each trial type j (win or loss trial). Modelling win and loss trials separately was required to observe valence-specific effects. Regarding the choice to have separate win and loss parameters is supported by patterns observed in non-model optimal choice behaviour, specifically the group x valence interaction. If parameters were combined, we would not be able to detect a valence-specific effect.

As per previous work [10], the learning expectation at initial state $Q_{0(s)}$ was set to 0.5. For each trial, the unchosen option $Q_{t(s')}$ was reciprocally updated based on the counterfactual outcome of the chosen symbol. Q values of the first two paired stimuli were aggregated to provide a value of choice probability via the following softmax function:

$$P_{t(s)} = \frac{1}{1 + \exp^{\left(Q_{t(s)} - Q_{t(s')}\right)}}$$

Each parameter was estimated by calculating posterior probability of the two trial paradigms followed by obtaining the expected value of each marginal likelihood parameter. Learning rate determines the slope of the learning curve, while outcome sensitivity determines the asymptote [10]. The outcome sensitivity parameter was sampled in discretised log space (110x100 grid), while the learning rate parameter was sampled in discretised logit space (110x100 grid). All task trials (30 trials per trial type) were fit to the model, and the parameter estimates were averaged over the three task blocks. Each model parameter was log transformed and then inferentially analysed using ANCOVA and EMM approaches.

Synthetic learning curves were simulated from choice probabilities generated during the model-fitting process for participant data. Comparing the real learning curve (Fig. 2B, main paper) with the synthetic one (Supplementary Fig. 1), the same pattern of divergence in learning during loss trials is observable between allocation groups. For parameter recovery, synthetic choice behaviour was simulated across a range of outcome sensitivity (0.5 – 19.5) and learning rate (0.05 – 0.95) values using a deterministic stimuli schedule from the task. This synthetic data was fitted to the model for ten permutations, and the recovered parameters were then compared to the true values used to generate them (Supplementary Fig. 2). As can be seen, all model parameters recover well unless outcome sensitivity is very high or very low.

An alternative model was considered which included inverse decision temperature β and learning rate (for both trial types). For this model, β was estimated at within the softmax function where it regulates how much the likelihood of the participant choosing shape s, $P_{t(s)}$, is influenced by differences in Q values. While outcome sensitivity ρ and inverse decision temperature β act on distinct parts of decision-making and learning throughout the task, within this model they are mathematically redundant [11], as demonstrated in Supplementary Fig. 3. As a result, we used prior evidence about the serotoninergic system to decide the most appropriate parameter to describe potential patterns in the behavioural data. Neurobiological evidence correlates neural burst firing in amygdalae 5-HT neurons to negative prediction errors during punishment, reflecting a neural signature of outcome sensitivity [12,13] . In contrast, there is no literature which suggests a link between 5-HT neuronal activity and choice stochasticity during model-free learning. This prior evidence guided choice to use a computational model which contained an outcome sensitivity and learning rate parameters to analyse task data.

In addition, we considered an alternative version of the outcome sensitivity model without reciprocal value updating (i.e., values were only updated for the chosen stimulus within each pair, not the unchosen one). We ultimately decided to retain the reciprocal-updating version of the model in the primary analysis, as this resulted in better performance within model validation by Halahakoon *et al.* [10]. Compared with the reciprocal updating model, the non-reciprocally updating

model performed equally well in explaining patterns in task behaviour (see Supplementary Results; Supplementary Fig. 13).

Signal Detection Theory Indices and Computational Drift Diffusion Modelling – Affective Interference Go/No-Go Task

Signal detection theory indices were derived using behavioural data from the Affective Interference Go/No-Go task. Required variables were calculated from task data: "Hit" (Correct 'Go' response), "Miss" (Missed 'Go' response), "False alarm" (Incorrect response to 'No-go' trial), and "Correct Rejection" (Correctly missed response to 'no-go' trial). These four variables were processed through the R package '*Psycho*' [14], and produced three indices (decision criterion [β], sensitivity index [d'] and alternate decision criterion [c]) which were log transformed. Each index was calculated using the following algorithms [15] :

$$d' = qnorm(hit) - qnorm(fa)$$

$$\beta = \exp\left(-\frac{zhr^2}{2} + \frac{zfar^2}{2}\right)$$

$$c = \frac{\left(M_{signal} - M_{noise}\right)}{\sigma}$$

While signal detection theory provides an estimate of signal discriminability between the groups, we decided to also fit observed behaviour to drift diffusion models (DDMs) to further understand behavioural patterns observed in the non-model data (i.e., differences in choice impulsivity across task conditions). The DDM provides a computational, mechanistic account of the evidence accumulation process during the task. We used a previously published, publicly available DDM approach for Go/No-Go data that relies on '*PyMC*' and '*HDDM*' Python packages [16–19], which resulted in high parameter recovery. Three models were fit to three task conditions: control (trials where no emotional distractors were present), positive interference (trials where happy distractor stimuli were present), and negative interference (trials where negative distractor stimuli were present). The model approach used assumed trials were independent of each other [17,20], and therefore the sequence of trials was assumed to be irrelevant to the evidence accumulation process. Participant data was fit using the gsquare approach which relies on maximum likelihood estimation, and is described as follows [17]:

$$G^2 = 2\,\Sigma\left(O \ln\left(\frac{O}{E}\right)\right)$$

For this approach, response time distributions were divided into five quantiles (10th, 30th, 50th, 70th and 90th), and were fed into gsquare along with associated choice behaviour. As the gsquare approach does not use hierarchical Bayesian fitting, the conditional independence assumption does not apply. The following model parameters were fit within the DDM: boundary separation (a), initial choice bias ($y(0) = z \cdot a$), non-decision time (T_{er}), drift rate (v) and drift criterion constant (dc). Initial choice bias and drift criterion were fixed per the previous approach [16]. All parameters were sampled in unconstrained logit space. The model is described using the following stochastic differential equation:

$$\Delta y = s \cdot v \cdot \Delta T_{er} + dc \cdot \Delta T_{er} + N(0, c^2 \cdot \Delta T_{er})$$

Model specifications without modification were adapted from a previously validated approach [16,17]. While alternative specifications and subsequent model comparison were considered, it was decided that use of a previously validated approach would offer more benefits (e.g., avoidance of intra-study bias generated from reparameterising the model for a specific set of data) and demonstrates the reliability and generalisability of the model [21,22]. Moreover, there was a close match between observed task data and synthetic data derived from the model fitting process for participant data (Supplementary Fig. 4A). For parameter recovery, data simulations of ~15000 trials per participant were performed using the Markov chain Monte Carlo method [23]. True values for the model were re-estimated without modifying model specifications to reflect the shortened response window within the task. Unlike parameter recovery for the reinforcement learning model, parameter recovery for the DDM was undertaken with reference to individual true parameter values, due to unrestricted space sampling and greater model complexity (*i.e.,* five model parameters). All model parameters were recoverable from synthetic data generated using true parameter estimates, as detailed in Supplementary Fig. 4B. All model parameters were log transformed and then inferentially analysed using baseline-adjusted ANCOVA and EMM approaches.

Data Collection and Analysis Software

For data collection, the following software packages were used: MATLAB 2021a; Psychtoolbox 3; PsychoPy 2021.1.4; Anaconda 2021.4; Presentation (Neurobs) 23.0; Qualtrics Surveys.

For data analysis, the following software packages were used: R (4.3.1); MATLAB (R2022a); Python (version 3.8.8); Docker 4.22.0. The following R packages were utilised: *dplyr* (1.1.2), *tidyverse* (2.0.0), *gtools* (3.9.4), *knitr* (1.42), *data.table* (1.14.8), *ggplot2* (3.4.2), *car* (3.1-2), *ggbeeswarm* (0.7.2), *ggrepel* (0.9.3), *readxl* (1.4.2), *openxlsx* (4.2.5.2), *ggpubr* (0.6.0), *rstatix* (0.7.2), "*ez*" (4.4-0), *ggsignif* (0.6.4), *RColorBrewer* (1.1-3), *emmeans* (1.8.5), *plotrix* (3.8-2), *sdamr* (0.2.0), *cowplot* – (1.1.1), *psycho* (0.6.1), *ggridges* (0.5.4), *viridis* (0.6.4), *ggstance* (0.3.6), *ggdist* (3.3.0), *gghalves* (0.1.4), *ggpp* (0.5.4), lme4 (1.1-33), *stringr* (1.5.0), *effectsize* (0.8.6), *lmerTest* (3.1-3).

All required Python dependencies are included within the Docker image: hcp4715/hddm:0.8. No additional MATLAB packages required.

Supplementary Note 1

Salivary Cortisol Analysis

Mean concentration of salivary cortisol (µg/dL) was analysed throughout the initial dose period (Supplementary Table 2; Supplementary Fig. 5). Before initial dose, salivary cortisol was at its lowest across the fenfluramine (mean = 0.12 ± 0.09) and placebo (mean = 0.13 ± 0.06) groups. One hour post-dose, salivary cortisol peaked in the fenfluramine (mean = 0.15 ± 0.10) and placebo (mean = 0.16 ± 0.12) groups. Three hours post-dose, salivary cortisol was sustained in the fenfluramine group (mean = 0.15 ± 0.09) but reduced in the placebo group (mean = 0.14 ± 0.05). In a baseline-adjusted ANCOVA analysis (T_0 as baseline; T_2 as follow-up) there was no significant main effect of group on salivary cortisol concentration at the final time-point ($F[1,50] = 0.02$, $p = 0.89$). In a linear mixed effects model, neither treatment allocation ($\beta = 0.01$, 95% CI [-0.03, 0.06], $t(112) = 0.54$, $p = 0.59$) significantly explained variance in a model of change in salivary cortisol before and after initial dose.

Supplementary Note 2

Self-report measures

There was no significant group difference across most self-report ratings of cognition, affect and mood at follow-up (see Table 1 (main paper); Supplementary Fig. 6). There was a group effect on

negative PANAS items at follow-up (ANCOVA main effect: F[1,38] = 5.00, p = 0.03, η_p^2 = 0.12 [0.00, 0.32]), however the EMM analysis did not indicate a significant between-groups difference (EMM = -0.75 ± 0.48, *p* = 0.12). There were no significant group effects for individual side effects items at follow-up (see Supplementary Table 3). In the longitudinal modelling analysis, there was no significant effect of group across daily ratings for VAS and side effects items (see main paper, Table 2; Supplementary Figs. 8-9). There was no significant effect of gender observed across all self-reported measures of cognition, affect, mood and side effects (Supplementary Table 7). Baseline data for each self-report measure is detailed in Supplementary Fig. 7. Due to survey software issues, self-report measure data was missing at random from both time points.

Supplementary Note 3

Probabilistic Instrumental Learning Task

Despite more optimal performance during loss trials in the placebo group, groups did not differ in terms of total money earned at follow-up (main effect of group ANCOVA: F[1,50] = 2.05, *p* = 0.16) (Supplementary Fig. 12A-B). As both groups performed equally well in win trials, it is likely that the decrease in optimal choices during loss was not enough to drive a statistically significant difference in total money earned.

Analysis of a version of the RL model without reciprocal value updating (i.e., values were only updated for the chosen stimulus within each pair, not the unchosen one) was undertaken. This model produced similar results to the reciprocal-updating model, where SSRA allocation reduced outcome sensitivity (ρ) within loss trials only (group x task condition: F[1,50] = 4.91, *p* = 0.03; win trials EMM = 0.10 ± 0.36, *p* = 0.78; loss trials EMM = -0.77 ± 0.36, *p* = 0.03), and learning rate (α) for both conditions did not significantly vary across groups (group x task condition: F[1,50] = 0.35, *p* = 0.56; main effect of group: F[1,50] = 0.95, *p* = 0.33) (Supplementary Fig. 13).

Across all metrics (non-model and computational), there were no significant effects of gender identified for this task (Supplementary Table 5). Baseline data for each task outcome is detailed in Supplementary Fig. 11A-D.

Supplementary Note 4

Affective Interference Go/No-Go Task

There was no significant interaction of group and set-shifting (rules changing or remaining the same across blocks) on response time via ANCOVA (F[1,47] = 0.03, *p* = 0.86) or response inhibition (F[1,47] = 0.06, *p* = 0.81), while there was a simple effect of set-shifting on response time only (F[1,347] = 5.18, *p* = 0.02, η_p^2 = 0.02 [0.00, 0.05).

In the analysis of signal detection theory indices, SSRA allocation resulted in more conservative responses (bias index log β) across all task conditions (ANCOVA main effect of group: F[1,47] = 12.67, *p* < 0.001, η_p^2 = 0.14 [0.00, 0.36]; all conditions EMM = 0.24 ± 0.08, *p* < 0.01, *d* = 0.35 [0.12, 0.58])). Further, while there was a main effect of group on sensitivity index d' (ANCOVA main effect of group: F[1,47] = 7.01, *p* = 0.01, η_p^2 = 0.08 [0.00, 0.28]), the follow-up EMM analysis did not indicate a significant between-groups difference (EMM = 0.15 ± 0.08, *p* = 0.06).

From the computational drift diffusion analysis (Supplementary Fig. 17), ANCOVA modelling revealed no significant effect of group on further model parameters: drift rate (v) (F[1,47] = 0.92, *p* = 0.34), boundary separation (a) (F[1,47] = 1.15, *p* = 0.29), and drift bias (dc) (F[1,47] = 0.10, *p* = 0.75). There was a treatment by group interaction for non-decision time (T_{er}) (F[2,95] = 3.71, p = 0.03, η_p^2 = 0.09 [0.00, 0.20]), but no significant main effect of group (F[1,47] = 0.02, *p* = 0.90). In a sensitivity analysis

of non-decision time in the SSRA group, there was a main effect for valence when isolating aversive vs control conditions (ANCOVA main effect: $F[1,22] = 5.72$, $p = 0.03$, $\eta_p^2 = 0.21$ [0.00, 0.47]) which was not observed in the placebo group (see Supplementary Table 9). However, post-hoc EMM analyses found no significant group differences in non-decision time across each task condition (aversive interference EMM = -0.01 ± 0.01, $p = 0.61$; positive interference EMM = -0.01 ± 0.01, $p = 0.87$; control condition EMM = 0.01 ± 0.01, $p = 0.31$). Baseline data for each task outcome (non-model and computational) is detailed in Supplementary Figs. 16A-E and 18.

There were no significant gender-related effects across nearly all task outcomes (non-model and computational), as shown in Supplementary Table 5. However, there was an effect of gender on the signal discriminability d' index from the signal detection theory model (main effect of gender: $F[1,45] = 20.81$, $p < 0.001$, $\eta_p^2 = 0.01$ [0.00, 0.15]; female vs male EMM = -0.26 ± 0.08, $p < 0.01$, d = -0.69 [-1.11, -0.27]), but no significant interaction between group and gender was observed (group x gender ANCOVA: $F[1,45] = 0.07$, $p = 0.79$). Similarly, there were differences across self-identified gender groups for β decision criterion (main effect of gender: $F[1,45] = 16.98$, $p < 0.001$, $\eta_p^2 = 0.03$ [0.01, 0.09]; female vs male EMM = -0.26 ± 0.08, $p < 0.01$, d = -0.39 [-0.63, -0.15]), but no significant interaction between group and gender was observed (group x gender ANCOVA: $F[1,45] = 0.35$, $p = 0.56$).

Supplementary Note 5

Rey Auditory Verbal Learning Task – intrusions and repetitions

Baseline-adjusted ANCOVA found no significant main effect of group allocation on number of intrusions ($F[1,48] = 0.26$, $p = 0.61$) and repetitions ($F[1,48] = 0.02$, $p = 0.89$), while a significant main effect of trial type was observed on intrusions ($F[2,48] = 4.38$, $p = 0.01$, $\eta_p^2 = 0.01$ [0.00, 0.01]). and repetitions ($F[2,48] = 4.53$, $p = 0.01$, $\eta_p^2 = 0.01$ [0.00, 0.01]). Mean intrusions and repetitions across each trial type are detailed in Supplementary Fig. 19B and 19C, respectively. Baseline data for each task outcome is detailed in Supplementary Fig. 20.

There were no significant gender-related effects on total words recalled during the task (Supplementary Table 5), which is where group-level differences were observed (reported in the main manuscript). There was a main effect of gender for total repetitions ($F[1,46] = 8.74$, $p < 0.01$, $\eta_p^2 = 0.01$ [0.00, 0.07]), but post-hoc EMM between genders found no significant difference [female vs male EMM = -0.08 ± 0.08, $p = 0.29$). Similarly, there was a group x gender x task condition interaction observed for total repetitions ($F[4,1470] = 4.37$, $p < 0.01$, $\eta_p^2 = 0.01$ [0.00, 0.02]) and total intrusions ($F[4,1470] = 4.03$, $p < 0.01$, $\eta_p^2 = 0.01$ [0.00, 0.01]), however post-hoc EMM analyses showed no statistically significant differences across drug groups within each gender across each trial type (Supplementary Table 6). It is important to interpret these gender x treatment x task condition interactions cautiously, as such complicated multi-level models are likely insufficiently powered by the study sample size.

Supplementary Note 6

Verbal n-Back Task

There was a main effect of n-back load (0-, 1-, 2-, or 3-back) on accuracy for targets (ANCOVA $F[3,143] = 63.68$, $p < 0.001$, $\eta_p^2 = 0.52$ [0.41, 0.60]) and response time (ANCOVA $F[3,143] = 43.34$, $p < 0.001$, $\eta_p^2 = 0.19$ [0.08, 0.29]). Across all metrics, there were no significant effects of gender identified for this task (Supplementary Table 5). Baseline data for each task outcome is detailed in Supplementary Fig. 21A-B.

Supplementary Note 7

Oxford Memory Test

Baseline-adjusted ANCOVA found no significant main effect of allocation on 7 of 8 metrics of visuospatial working memory performance on the OMT at follow-up, as summarised in Supplementary Table 4 and Supplementary Fig. 22. A significant main effect of group allocation on identification time at follow-up was observed (ANCOVA: $F[1,47] = 5.56$, $p = 0.02$; $\eta_p^2 = 0.01$ [0.00, 0.06]), however a post-hoc EMM analysis found no significant difference in identification time between allocation groups (EMM = 0.16 ± 0.09, $p = 0.09$). Across all metrics, there were no significant effects of gender identified for this task (Supplementary Table 5). Baseline data for each task outcome is detailed within Supplementary Table 6 and Supplementary Fig. 23.

Supplementary Note 8

Contextual cueing task

Baseline-adjusted ANCOVA found no significant main effect of group on accuracy difference (cued – novel trials) in the contextual cueing task at the follow-up (main effect of group: $F[1,50] = 0.96$, $p = 0.332$; group x task stage ANCOVA: $F[1,50] = 0.37$, $p = 0.55$) (Supplementary Fig. 24A). Similarly, no significant main effect of group allocation was observed on response time difference (cued – novel trials) during the task (main effect of group: $F[1,50] = 0.04$, $p = 0.84$; group x task stage ANCOVA: $F[1,50] = 0.32$, $p = 0.58$), while a simple main effect of task stage (first half/second half) was observed (ANCOVA: $F[1,50] = 17.10$, $p < 0.001$; $\eta_p^2 = 0.26$ [0.08, 0.44]). Mean response time for each allocation group across novel or cued stimuli is detailed in Supplementary Fig. 24B). Across all metrics, there were no significant effects of gender identified for this task (Supplementary Table 5). Baseline data for each task outcome is detailed in Supplementary Fig. 25A-B.

Supplementary Tables

Supplementary Table 1. Demographic characteristics across allocation groups

	Fenfluramine (*n*=26)	Placebo (*n*=27)	Inferential analysis [a]
Age (Years), M (S.D.)	20.19 (1.36)	20.15 (1.29)	0.82
Gender, *N* male:female	10:16	10:16	1.00
Body mass index, M (S.D.)	22.36 (3.48)	22.75 (2.57)	0.77
Contraceptive use, yes:no	6:8	9:7	0.65
Native language, *N*			0.67
English	19	20	
Chinese	3	3	
Other	4	3	
Time in education (Years), M (S.D.)	15.17 (1.22)	15.27 (1.73)	0.83
Highest Educational Attainment, *N*			0.79
High School / Sixth form	18	20	
Undergraduate degree	7	6	
Postgraduate degree	1	0	
Not applicable	0	1	
Family History – Mental Health, yes:no	5:21	8:18	0.49

[a] Values represent significance values inferential analyses. These values pertain to Welch's two Sample t-test where differences between group means were analysed, and Pearson's Chi-squared where differences between frequency/ratio distributions were analysed.

Supplementary Table 2. Mean concentration (µg/dL) salivary cortisol throughout the initial dose period

	Fenfluramine (n=26)	Placebo (n=27)
**OMT Domain**	**Mean ± SD**	**Mean ± SD**
T_0: Before Dose	0.12 ± 0.06	0.13 ± 0.06
T_1: 1 hour post-dose	0.15 ± 0.10	0.16 ± 0.12
T_2: 3 hours post-dose	0.15 ± 0.09	0.14 ± 0.05

Supplementary Table 3. Side effects profile for allocation groups – descriptive statistics and inferential analysis

Side effect domain	Fenfluramine (*n*=26) M (S.D.)	Placebo (*n*=27) M (S.D.)	Inferential analysis [a]	
			F-statistic [df]	*p*
**Appetite, decreased**				
Baseline, M (S.D.)	0.19 (0.40)	0.35 (0.71)	--	--
Follow-up, M (S.D.)	0.52 (0.82)	0.16 (0.37)	3.96 [1,47]	0.052
**Appetite, increased**				
Baseline, M (S.D.)	0.15 (0.37)	0.17 (0.39)	--	--
Follow-up, M (S.D.)	0.04 (0.20)	0.12 (0.33)	2.16 [1,47]	0.148
**Drowsiness/Fatigue**				
Baseline, M (S.D.)	0.42 (0.70)	1.13 (0.82)	--	--
Follow-up, M (S.D.)	0.68 (0.69)	0.28 (0.54)	3.28 [1,47]	0.076
**Insomnia**				
Baseline, M (S.D.)	0.00 (0.00)	0.09 (0.29)	--	--
Follow-up, M (S.D.)	0.12 (0.44)	0.16 (0.37)	0.49 [1.47]	0.489
**Sexual side effects**				
Baseline, M (S.D.)	0.00 (0.00)	0.09 (0.42)	--	--
Follow-up, M (S.D.)	0.12 (0.33)	0.04 (0.20)	0.21 [1,47]	0.649
**Sweating**				
Baseline, M (S.D.)	0.04 (0.20)	0.09 (0.29)	--	--
Follow-up, M (S.D.)	0.04 (0.20)	0.00 (0.00)	0.98 [1,47]	0.327
**Tremors**				
Baseline, M (S.D.)	0.00 (0.00)	0.00 (0.00)	--	--
Follow-up, M (S.D.)	0.00 (0.00)	0.08 (0.28)	2.09 [1,47]	0.155
**Agitation**				
Baseline, M (S.D.)	0.04 (0.20)	0.13 (0.34)	--	--
Follow-up, M (S.D.)	0.16 (0.47)	0.04 (0.20)	1.54 [1,47]	0.221
**Anxiety**				
Baseline, M (S.D.)	0.15 (0.37)	0.22 (0.42)	--	--
Follow-up, M (S.D.)	0.00 (0.00)	0.08 (0.40)	1.85 [1,47]	0.180
**Diarrhoea**				
Baseline, M (S.D.)	0.00 (0.00)	0.00 (0.00)	--	--
Follow-up, M (S.D.)	0.28 (0.84)	0.04 (0.20)	1.92 [1,47]	0.172
**Dry Mouth**				
Baseline, M (S.D.)	0.08 (0.27)	0.04 (0.21)	--	--
Follow-up, M (S.D.)	0.24 (0.44)	0.12 (0.33)	0.00 [1,47]	1.000
**Indigestion**				
Baseline, M (S.D.)	0.00 (0.00)	0.04 (0.21)	--	--
Follow-up, M (S.D.)	0.00 (0.00)	0.08 (0.28)	2.06 [1,47]	0.158
**Nausea**				
Baseline, M (S.D.)	0.04 (0.17)	0.09 (0.29)	--	--
Follow-up, M (S.D.)	0.16 (0.55)	0.04 (0.20)	1.02 [1,47]	0.318
**Upset stomach**				
Baseline, M (S.D.)	0.08 (0.27)	0.09 (0.29)	--	--
Follow-up, M (S.D.)	0.16 (0.55)	0.12 (0.33)	0.10 [1,47]	0.760

[a] Inferential analysis via baseline-adjusted ANCOVA model (two-tailed) across allocation groups (active vs placebo).

Supplementary Table 4. Oxford Memory Test (visual working memory) ANCOVA summary with main effect of treatment allocation

	Fenfluramine (n=24)	Placebo (n=24)			
Follow-up data	**Mean ± SD**	**Mean ± SD**	**F-Statistic** [a]	**Degrees of freedom**	***P*-Value**
Absolute Error	74.33 ± 44.94	79.06 ± 55.36	0.40	1,47	0.53
Misbinding value	0.07 ± 0.09	0.09 ± 0.10	0.08	1,47	0.79
Guessing value	0.09 ± 0.08	0.08 ± 0.07	0.19	1,47	0.67
Targeting value	0.85 ± 0.15	0.85 ± 0.15	0.03	1,47	0.86
Identification time	1.64 ± 0.70	1.48 ± 0.61	5.56	1,47	0.02
Localisation time	3.30 ± 1.09	3.20 ± 1.00	0.43	1,47	0.52
Proportion correct	0.96 ± 0.05	0.96 ± 0.05	0.00	1,47	1.00
Imprecision value	50.67 ± 17.49	51.49 ± 19.44	0.08	1,47	0.78
Baseline data					
Absolute Error	76.00 ± 54.65	85.34 ± 57.83	--	--	--
Misbinding value	0.07 ± 0.10	0.09 ± 0.11	--	--	--
Guessing value	0.10 ± 0.09	0.11 ± 0.07	--	--	--
Targeting value	0.83 ± 0.17	0.81 ± 0.16	--	--	--
Identification time	1.72 ± 0.80	1.60 ± 0.79	--	--	--
Localisation time	3.59 ± 1.09	3.69 ± 1.12	--	--	--
Proportion correct	0.96 ± 0.05	0.96 ± 0.05	--	--	--
Imprecision value	48.59 ± 16.07	51.25 ± 17.76	--	--	--

[a] via baseline-adjusted ANCOVA type II modelling (two-tailed).

Supplementary Table 5. Analysis of self-identified gender effects on task battery data at follow-up

Task Battery Data	Fenfluramine [a]		**Placebo** [a]		**F-Statistic** [b]	**DOF**	***P*-Value**
	Female	*Male*	*Female*	*Male*			
	**Mean ± SD**	**Mean ± SD**	**Mean ± SD**	**Mean ± SD**			
**Probabilistic Instrumental Learning Task**							
Optimal choices							
Main effect: gender	42.84 ± 12.78	45.25 ± 12.99	48.00 ± 11.27	47.36 ± 10.83	0.11	1,48	0.74
Gender x grp int.	--	--	--	--	0.16	1,48	0.69
Gender x grp x task condition int.	--	--	--	--	0.80	2,48	0.45
Response time (ms)							
Main effect: gender	1110.62 ± 460.96	993.73 ± 414.01	888.49 ± 291.77	1004.32 ± 329.68	0.01	1,48	0.97
Gender x grp int.	--	--	--	--	1.19	1,48	0.28
Gender x grp x task condition int.	--	--	--	--	1.07	2,48	0.35
Outcome sensitivity (Log)							
Main effect: gender	1.27 ± 2.06	1.23 ± 2.07	1.45 ± 1.85	1.96 ± 1.76	0.19	1,48	0.67
Gender x grp int.	--	--	--	--	0.04	1.48	0.84
Gender x grp x task condition int.	--	--	--	--	1.22	2,48	0.31
Total Money Earned (£)							
Main effect: gender	5.04 ± 2.42	5.90 ± 1.88	5.74 ± 2.02	6.29 ± 2.06	1.84	1,48	0.18
Gender x grp int.	--	--	--	--	0.20	1,48	0.66
Learning Rate (Log)							
Main effect: gender	-2.04 ± 0.99	-1.97 ± 1.22	-2.26 ± 1.06	-2.15 ± 1.23	0.58	1,48	0.45
Gender x grp int.	--	--	--	--	2.52	1,48	0.12
Gender x grp x task condition int.	--	--	--	--	0.47	2,48	0.63
**Affective Go/No-Go Task**							
'Go' Trial accuracy							
Main effect: gender	90.94 ± 5.54	92.36 ± 6.14	91.49 ± 8.29	94.28 ± 3.37	2.66	1,45	0.11
Gender x grp int.	--	--	--	--	0.08	1,45	0.78
Gender x grp x task condition int.	--	--	--	--	1.12	4,91	0.36
'No-go' trial accuracy (response inhibition)							
Main effect: gender	80.10 ± 14.52	80.31 ± 12.39	70.42 ± 19.33	70.58 ± 15.10	0.01	1,45	0.95
Gender x grp int.	--	--	--	--	0.11	1,45	0.75
Gender x grp x task condition int.	--	--	--	--	0.70	4,91	0.59
Gender x grp x set-shifting int.	--	--	--	--	0.11	2,345	0.75
Response time (ms)							
Main effect: gender	292.06 ± 18.94	287.60 ± 10.65	273.71 ± 19.64	273.71 ± 19.64	0.37	1,45	0.55
Gender x grp int.	--	--	--	--	0.20	1,45	0.66
Gender x grp x task condition int.	--	--	--	--	0.50	4,91	0.74
Decision Criterion (c; log) – SDT analysis							
Main effect: gender	0.52 ± 0.22	0.50 ± 0.12	0.45 ± 0.21	0.42 ± 0.16	1.55	1,45	0.22
Gender x grp int.	--	--	--	--	0.04	1,45	0.85
Gender x grp x task condition int.	--	--	--	--	0.56	4,241	0.85
Decision Criterion (β; log) – SDT analysis							
Main effect: gender	-0.55 ± 0.78	-0.87 ± 0.63	-0.79 ± 0.59	-1.05 ± 0.61	16.98	1,45	< 0.001

Gender x grp int.	--	--	--	--	0.35	1,45	0.56
Gender x grp x task condition int.	--	--	--	--	0.83	4,241	0.51
Sensitivity index (d') – SDT analysis							
Main effect: gender	2.31 ± 0.54	2.63 ± 0.84	2.19 ± 0.70	2.40 ± 0.64	20.81	1,45	< 0.001
Gender x grp int.	--	--	--	--	0.07	1,45	0.79
Gender x grp x task condition int.	--	--	--	--	0.36	4,241	0.84
Initial Choice Bias (Log) – DDM							
Main effect: gender	-1.35 ± 0.74	-1.03 ± 0.32	-1.14 ± 0.51	-0.97 ± 0.24	3.29	1,45	0.08
Gender x grp int.	--	--	--	--	0.45	1,45	0.51
Gender x grp x task condition int.	--	--	--	--	0.72	4,91	0.58
Non-decision time (Log) – DDM							
Main effect: gender	0.17 ± 0.04	0.18 ± 0.02	0.17 ± 0.04	0.18 ± 0.02	1.19	1,45	0.28
Gender x grp int.	--	--	--	--	0.01	1,45	0.94
Gender x grp x task condition int.	--	--	--	--	2.51	2,91	0.09
Drift Rate (Log) – DDM							
Main effect: gender	2.66 ± 0.16	2.70 ± 0.15	2.64 ± 0.10	2.68 ± 0.07	2.59	1,45	0.12
Gender x grp int.	--	--	--	--	0.01	1,45	0.96
Gender x grp x task condition int.	--	--	--	--	0.85	4,91	0.50
Boundary separation (Log) – DDM							
Main effect: gender	-0.16 ± 0.35	0.21 ± 0.24	-0.23 ± 0.29	-0.29 ± 0.12	0.57	1,45	0.46
Gender x grp int.	--	--	--	--	0.00	1,45	0.98
Gender x grp x task condition int.	--	--	--	--	1.46	4,91	0.22
Drift Criterion/bias – DDM							
Main effect: gender	2.60 ± 0.14	2.53 ± 0.10	2.57 ± 0.11	2.55 ± 0.05	2.67	1,44	0.11
Gender x grp int.	--	--	--	--	0.58	1,44	0.45
Gender x grp x task condition int.	--	--	--	--	1.21	4,90	0.31
**Contextual Cueing Task**							
Accuracy difference							
Main effect: gender	-0.34 ± 2.52	0.70 ± 3.81	0.28 ± 2.70	1.41 ± 3.50	2.34	1,48	0.13
Gender x grp int.	--	--	--	--	0.01	1,48	0.95
Gender x grp x task condition int.	--	--	--	--	0.78	2,48	0.46
Response time difference							
Main effect: gender	-11.56 ± 39.08	-9.08 ± 36.70	-16.08 ± 31.28	-6.19 ± 30.45	0.74	1,48	0.83
Gender x grp int.	--	--	--	--	0.48	1,48	0.49
Gender x grp x task condition int.	--	--	--	--	1.38	2,48	0.26
**Oxford Memory Test**							
Absolute error							
Main effect: gender	80.77 ± 51.43	64.67 ± 31.76	76.25 ± 56.98	83.28 ± 54.00	0.35	1,45	0.53
Gender x grp int.	--	--	--	--	1.54	1,45	0.22
Misbinding value							
Main effect: gender	0.07 ± 0.10	0.05 ± 0.07	0.07 ± 0.10	0.07± 0.10	0.92	1,45	0.34
Gender x grp int.	--	--	--	--	2.17	1,45	0.15
Guessing value							
Main effect: gender	0.11 ± 0.09	0.07 ± 0.07	0.08 ± 0.08	0.09 ± 0.06	0.71	1,45	0.40
Gender x grp int.	--	--	--	--	0.60	1,45	0.44

Targeting value							
Main effect: gender	0.82 ± 0.16	0.88 ± 0.12	0.85 ± 0.15	0.84 ± 0.15	0.90	1,45	0.35
Gender x grp int.	--	--	--	--	1.20	1,45	0.28
Identification time							
Main effect: gender	1.56 ± 0.67	1.76 ± 0.73	1.49 ± 0.58	1.48 ± 0.67	1.91	1,45	0.17
Gender x grp int.	--	--	--	--	0.13	1,45	0.72
Localisation time							
Main effect: gender	3.31 ± 1.15	3.29 ± 1.01	3.27 ± 1.10	3.10 ± 0.79	0.36	1,45	0.55
Gender x grp int.	--	--	--	--	0.41	1,45	0.53
Proportion correct							
Main effect: gender	0.96 ± 0.05	0.97 ± 0.04	0.96 ± 0.05	0.96 ± 0.06	0.04	1,45	0.85
Gender x grp int.	--	--	--	--	0.29	1,45	0.60
Imprecision value							
Main effect: gender	50.27 ± 19.87	51.26 ± 13.62	48.26 ± 19.05	56.33 ± 19.49	2.21	1,45	0.14
Gender x grp int.	--	--	--	--	0.31	1,45	0.58
**Verbal n-back task**							
Target accuracy							
Main effect: gender	8.80 ± 1.01	8.84 ± 1.08	8.63 ± 1.11	8.80 ± 1.19	0.62	1,45	0.43
Gender x grp int.	--	--	--	--	0.63	1,45	0.43
Gender x grp x task condition int.	--	--	--	--	1.00	6,137	0.43
Response time (ms)							
Main effect: gender	622.58 ± 149.19	660.64 ± 231.46	659.03 ± 168.56	688.58 ± 216.80	1.34	1,45	0.25
Gender x grp int.	--	--	--	--	0.01	1,45	0.92
Gender x grp x task condition int.	--	--	--	--	1.99	6,137	0.07
**Auditory Verbal Learning Task**							
Total recall accuracy							
Main effect: gender	12.28 ± 2.70	12.32 ± 2.62	12.27 ± 2.75	12.18 ± 3.08	0.04	1,46	0.84
Gender x grp int.	--	--	--	--	0.28	1,46	0.60
Gender x grp x task condition int.	--	--	--	--	1.31	4,1470	0.26
Total recall repetitions							
Main effect: gender	0.19 ± 0.52	0.32 ± 1.12	0.14 ± 0.43	0.41 ± 0.78	8.74	1,46	< 0.01
Gender x grp int.	--	--	--	--	0.12	1,46	0.74
Gender x grp x task condition int.	--	--	--	--	4.37	4,1470	< 0.01
Total recall intrusions							
Main effect: gender	0.08 ± 0.32	0.13 ± 0.33	0.06 ± 0.23	0.13 ± 0.37	0.75	1,46	0.39
Gender x grp int.	--	--	--	--	0.04	1,46	0.83
Gender x grp x task condition int.	--	--	--	--	4.03	4,1470	< 0.01

[a] Gender allocation to SSRA fenfluramine was 16:10 (f:m), while allocation to placebo was 16:11 (f:m).

[b] via baseline-adjusted ANCOVA type II modelling.

Abbreviations: Grp = Group; DOF = Degrees of freedom.

Supplementary Table 6. Fenfluramine vs Placebo EMMs: investigation of Gender x Group x Task Condition interaction on the AVLT

AVLT Task Stage	Gender	EMM ± SE [a]	95% CI	*P*-Value
Learning trials – Repetitions	Female	0.17 ± 0.13	-0.09, 0.44	0.20
Learning trials – Repetitions	Male	0.12 ± 0.17	-0.21, 0.45	0.47
Distraction trials – Repetitions	Female	0.01 ± 0.05	-0.09, 0.12	0.84
Distraction trials – Repetitions	Male	-0.06 ± 0.07	-0.20, 0.07	0.33
Delayed recall trials – Repetitions	Female	0.01 ± 0.27	-0.52, 0.53	0.98
Delayed recall trials – Repetitions	Male	-0.64 ± 0.33	-1.29, 0.02	0.06
Learning trials – Intrusions	Female	0.00 ± 0.05	-0.10, 0.10	0.97
Learning trials – Intrusions	Male	0.09 ± 0.07	-0.04, 0.21	0.19
Distraction trials – Intrusions	Female	0.03 ± 0.02	-0.01, 0.07	0.15
Distraction trials – Intrusions	Male	0.02 ± 0.03	-0.03, 0.07	0.54
Delayed recall trials – Intrusions	Female	0.01 ± 0.10	-0.20, 0.21	0.94
Delayed recall trials – Intrusions	Male	-0.25 ± 0.13	-0.51, 0.00	0.053

[a] Post-hoc exploratory SSRA fenfluramine vs placebo EMM—adjusted for multiple comparisons via Bonferroni-Holm procedure.

Supplementary Table 7. Analysis of self-identified gender effects on questionnaire data at follow-up

Task Battery Data	Fenfluramine [a]		**Placebo** [a]		**F-Statistic** [b]	**DOF**	***P*-Value**
	Female	*Male*	*Female*	*Male*			
	**Mean ± SD**	**Mean ± SD**	**Mean ± SD**	**Mean ± SD**			
Spielberger Trait Anxiety Subscale							
Main effect: gender	33.33 ± 5.40	22.56 ± 6.25	22.17 ± 6.19	21.80 ± 4.08	0.94	1,34	0.34
Gender x grp int.	--	--	--	--	0.10	1,34	0.75
Spielberger State Anxiety Subscale							
Main effect: gender	1.00 ± 1.81	2.11 ± 1.76	2.42 ± 3.53	2.30 ± 2.06	0.44	1,34	0.51
Gender x grp int.	--	--	--	--	0.01	1,34	0.97
Beck Depression Inventory							
Main effect: gender	2.88 ± 3.10	3.44 ± 2.92	2.63 ± 3.10	3.90 ± 5.49	1.26	1,41	0.27
Gender x grp int.	--	--	--	--	2.13	1,14	0.15
Positive & Negative Affect Schedule - Negative							
Main effect: gender	10.77 ± 1.24	11.00 ± 1.31	11.33 ± 1.63	12.13 ± 2.53	0.58	1,36	0.45
Gender x grp int.	--	--	--	--	1.21	1,36	0.28
Positive & Negative Affect Schedule – Positive							
Main effect: gender	28.85 ± 7.96	25.63 ± 3.74	26.13 ± 7.87	26.38 ± 8.48	0.48	1,36	0.50
Gender x grp int.	--	--	--	--	0.01	1,36	0.94
Visual Analogue Scale - Negative							
Main effect: gender	141.38 ± 93.90	169.30 ± 75.80	148.69 ± 89.13	187.00 ± 91.61	2.87	1,44	0.10
Gender x grp int.	--	--	--	--	0.51	1,44	0.48
Visual Analogue Scale - Positive							
Main effect: gender	710.36 ± 108.67	682.40 ± 91.86	723.44 ± 110.39	657.55 ± 127.82	3.37	1,42	0.07
Gender x grp int.	--	--	--	--	2.00	1,45	0.16
Perceived Deficits Questionnaire							
Main effect: gender	11.20 ± 10.95	18.29 ± 17.811	13.31 ± 13.93	13.40 ± 12.39	1.73	1,35	0.20
Gender x grp int.	--	--	--	--	2.24	1,35	0.14
Side Effects Overall							
Main effect: gender	2.69 ± 3.36	2.22 ± 2.17	1.33 ± 2.85	2.18 ± 2.82	0.05	1,45	0.83
Gender x grp int.	--	--	--	--	1.88	1,45	0.18

[a] Gender allocation to SSRA fenfluramine was 16:10 (f:m), while allocation to placebo was 16:11 (f:m).

[b] via baseline-adjusted ANCOVA type II modelling.

Abbreviations: Grp = Group; DOF = Degrees of freedom.

Supplementary Table 8. Valence-specific contrasts for 'Go' trial response times in the Affective Interference Go/No-Go Task

	**F-Statistic** [a]	**Degrees of Freedom**	***P*-Value**
Fenfluramine Group (n=24)			
Aversive vs Control	4.87	1,22	0.04
Happy vs Control	3.05	1,22	0.10
Placebo Group (n=26)			
Aversive vs Control	1.95	1,24	0.28
Happy vs Control	1.00	1,24	0.33

[a] via baseline-adjusted ANCOVA type II modelling.

Supplementary Table 9. Valence-specific contrasts for non-decision time (DDM parameter) in the Affective Interference Go/No-Go Task

	**F-Statistic** [a]	**Degrees of Freedom**	***P*-Value**
Fenfluramine Group (n=24)			
Aversive vs Control	5.72	1,22	0.03
Happy vs Control	1.09	1,22	0.28
Placebo Group (n=26)			
Aversive vs Control	1.49	1,24	0.22
Happy vs Control	0.12	1,24	0.67

[a] via baseline-adjusted ANCOVA type II modelling.

Supplementary Figures

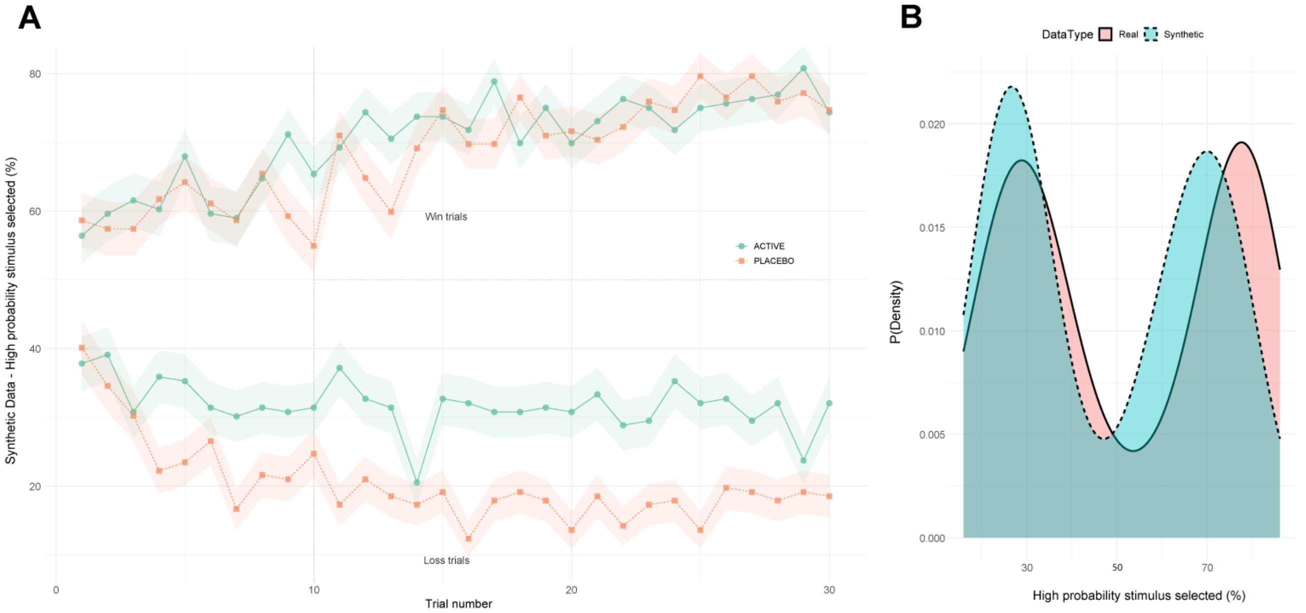

Supplementary Fig. 1. Model synthetic learning curve and comparison with observed task data. A. Synthetic learning curve derived from choice probabilities produced during the model-fitting process for participant data. Temporal learning patterns on the curve are split across each trial type (win or loss trials) across allocation groups at follow-up (see Figure 2B in the main paper for real data comparison). The shaded area around lines represents standard error. **B.** Probability density plot comparison between observed task data (red; continuous line) and synthetic data derived from the model fitting process for participant data (green; dashed line), using data from both study visits (baseline and follow-up). Synthetic data from panels **A** and **B** were derived from models fit to data from *N*=53 individuals.

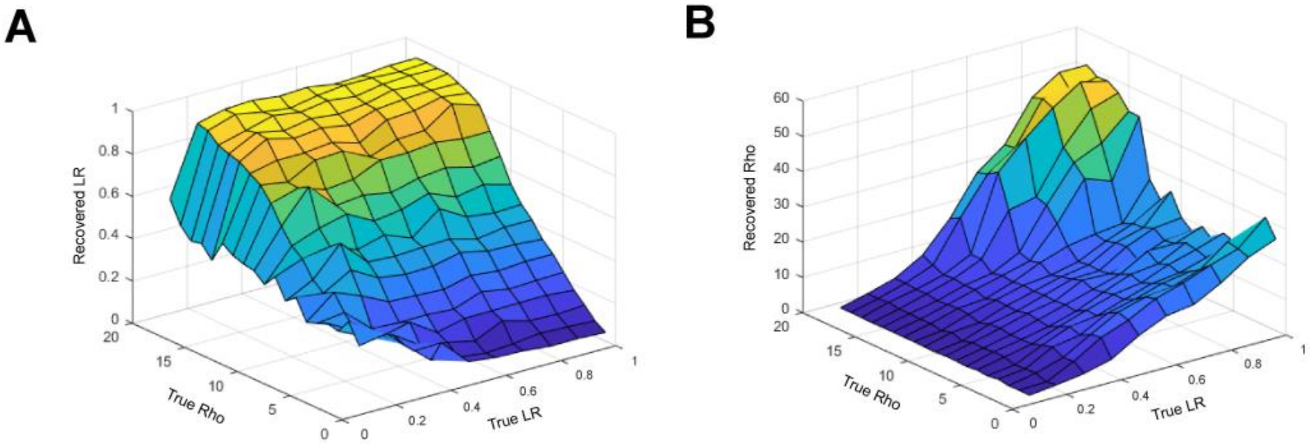

Supplementary Fig. 2. Reinforcement learning model parameter recovery. Synthetic choice behaviour was generated across a range of potential true parameter combinations, and then was fitted to the model to generate recovered parameter values. Depicted using a three-dimensional plot, Panel **A** shows the learning rate (LR) recovery for all trials. Panel **B** shows outcome sensitivity (rho) recovery for all trials. Learning rate was recovered across all combinations of true parameters, while rho was recoverable in most instances except where rho and LR were very high. Values in Panels **A** and **B** represent averaged values for each recovered parameter across *N*=100^3 simulations. The colour gradient in Panels **A** and **B** represent the magnitude or intensity of the values on the z-axis.

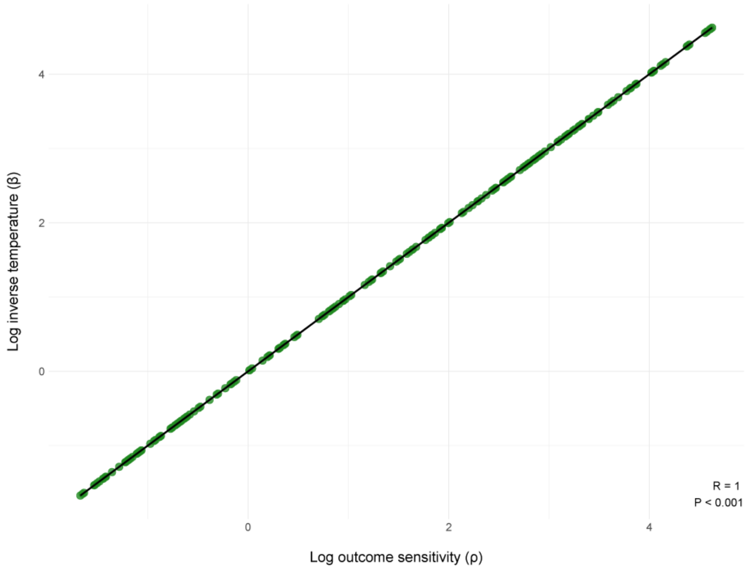

Supplementary Fig. 3. Relationship between each reinforcement learning model parameter, outcome sensitivity ρ and inverse temperature β. R and significance values (*P*) relate to two-tailed Pearson's product-moment correlation analysis on all task data at follow-up. Line fitted through data points using linear modelling. These parameters values were derived from models fit to data from *N*=53 individuals.

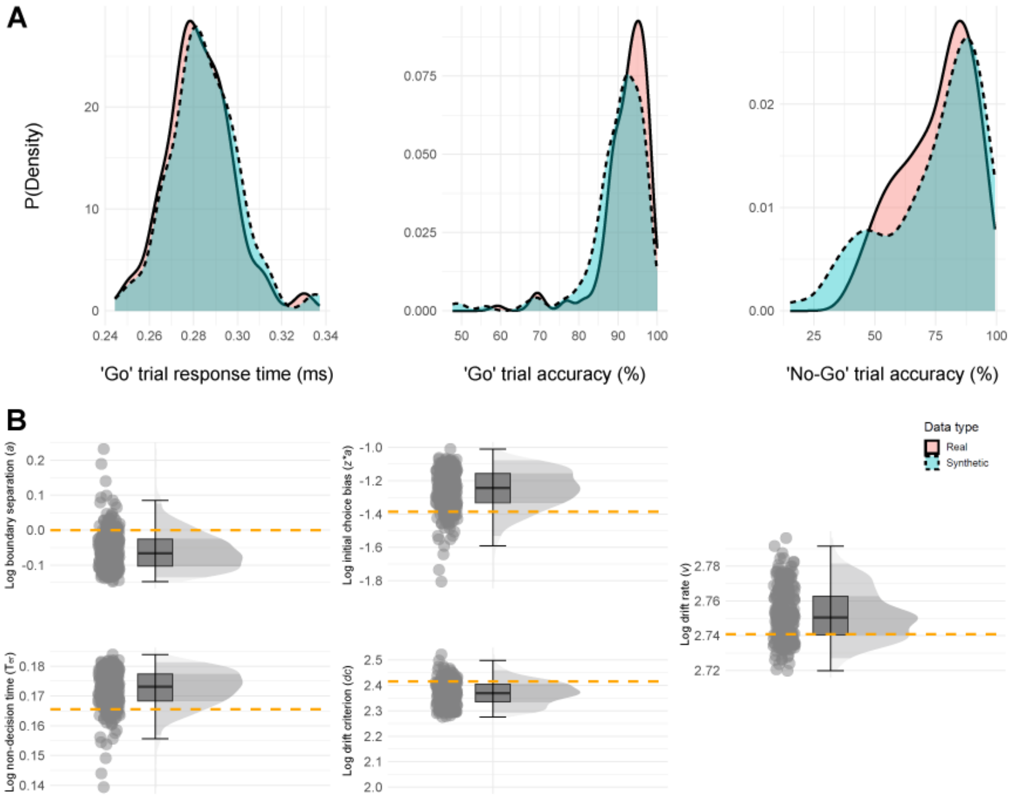

Supplementary Fig. 4. Drift Diffusion Model model-fit data simulation and parameter recovery. A. Probability density curve comparison between observed task data (red; continuous line) and synthetic data derived from the model fitting process for participant data (green; dashed line) using data from both study visits (baseline and follow-up). **B.** Recovered parameter values (log transformed) from synthetic data generated using true parameter estimates. True parameter values are denoted by the dashed orange line. Synthetic data from panel **A** were derived from models fit to data from *N*=53 individuals. Values in Panel **B** represent averaged values for each recovered parameter across *N*=795,000 trial simulations. Boxplots represent the interquartile range (IQR), while the central line depicts the median. The whiskers extend to approximately ± 1.5 times the IQR, encompassing the bulk of the data points; half-violin plots depict the data distribution.

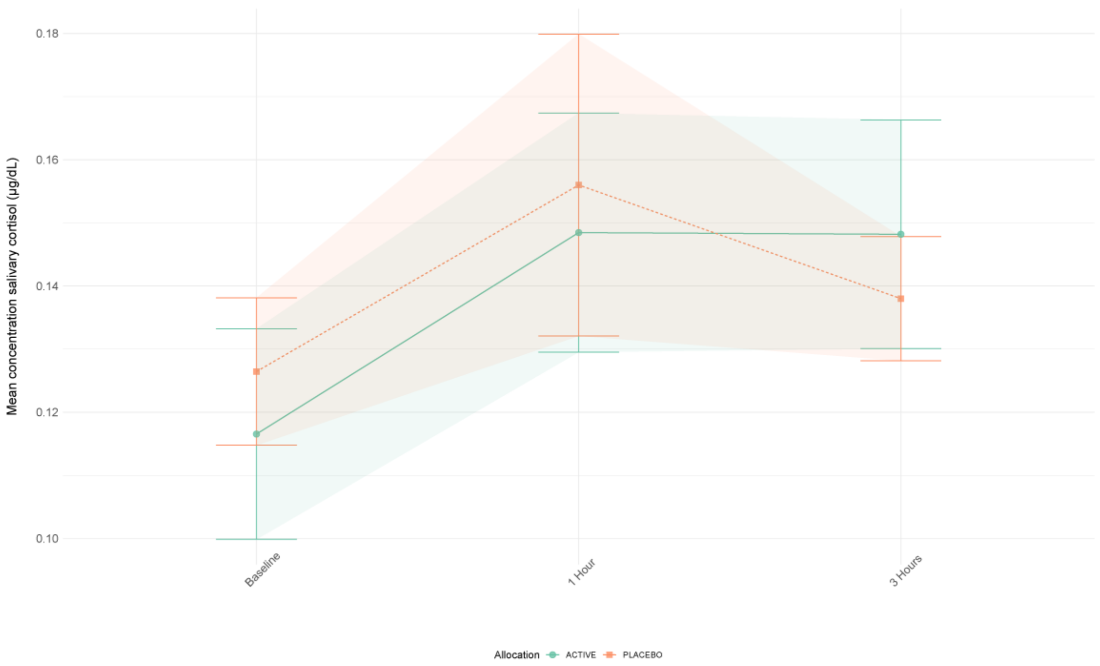

Supplementary Fig. 5. Cortisol concentration across groups during the initial dose period. Mean concentration salivary cortisol (µg/dL) across allocation groups during the initial dose period (Baseline [t_0], 1 hour post-dose [t_1], and 3 hours post-dose [t_2]). Cortisol data in this figure was collected from *N*=53 individuals across each time point; lines and plot points depict mean value, with error bars and shaded areas around each line depicting standard mean error.

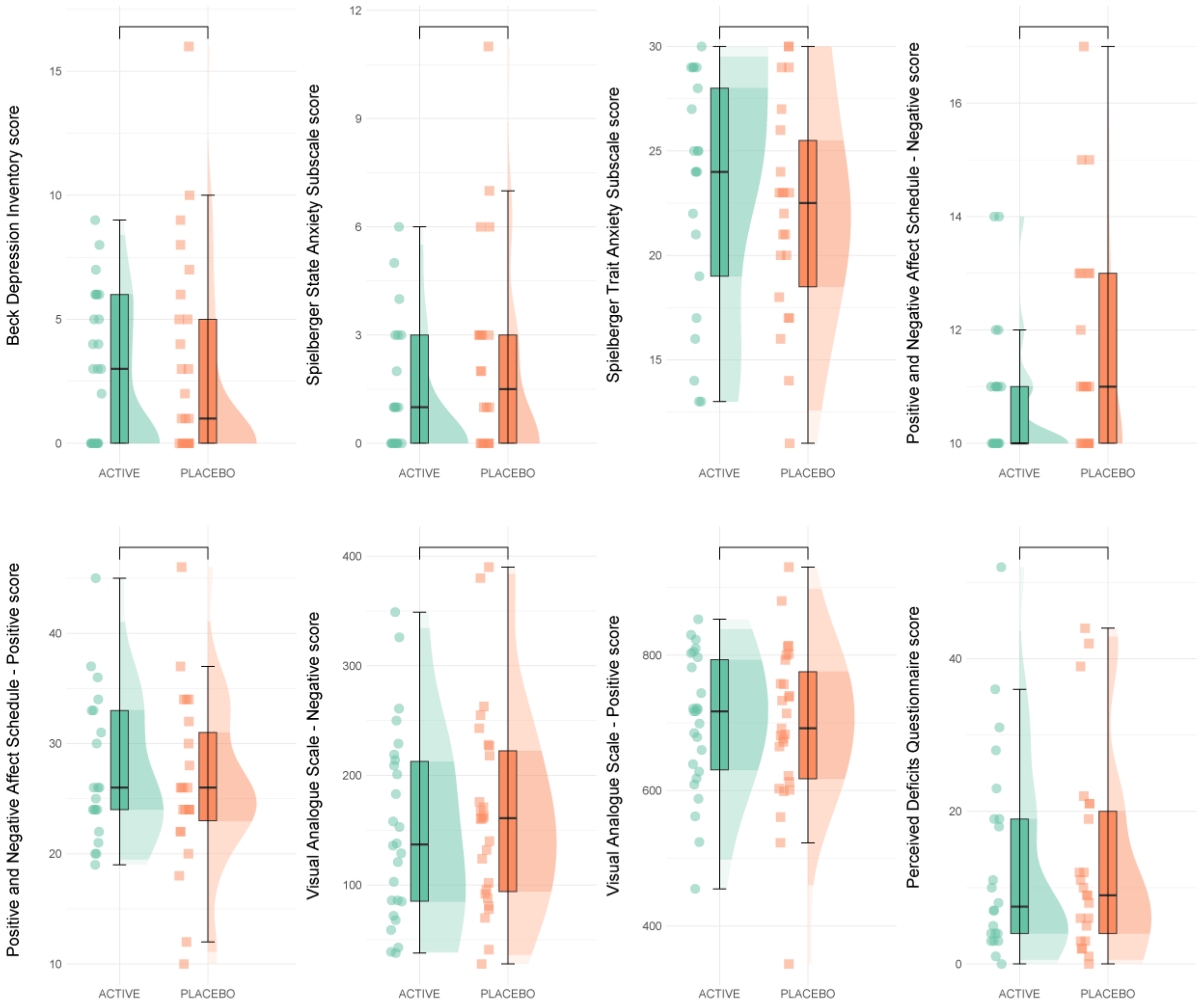

Supplementary Fig. 6. Self-reported Questionnaire measure scores at follow-up across allocation groups. At the final study visit, self-reported participant data was collected across multiple measures. Beck Depression Inventory analysis contained data from *N*=51 individuals. Spielberger subscales (trait/state) contained data from *N*=43 individuals. Positive and Negative Affect Schedule subscales (positive and negative items) contained data from *N*=44 individuals. Visual Analogue Scale subscales (positive and negative items) contained data from *N*=53 individuals. Perceived Deficits Questionnaire analysis contained data from *N*=45 individuals. Boxplots represent the interquartile range (IQR), while the central line depicts the median. The whiskers extend to approximately ± 1.5 times the IQR, encompassing the bulk of the data points; half-violin plots depict the data distribution.

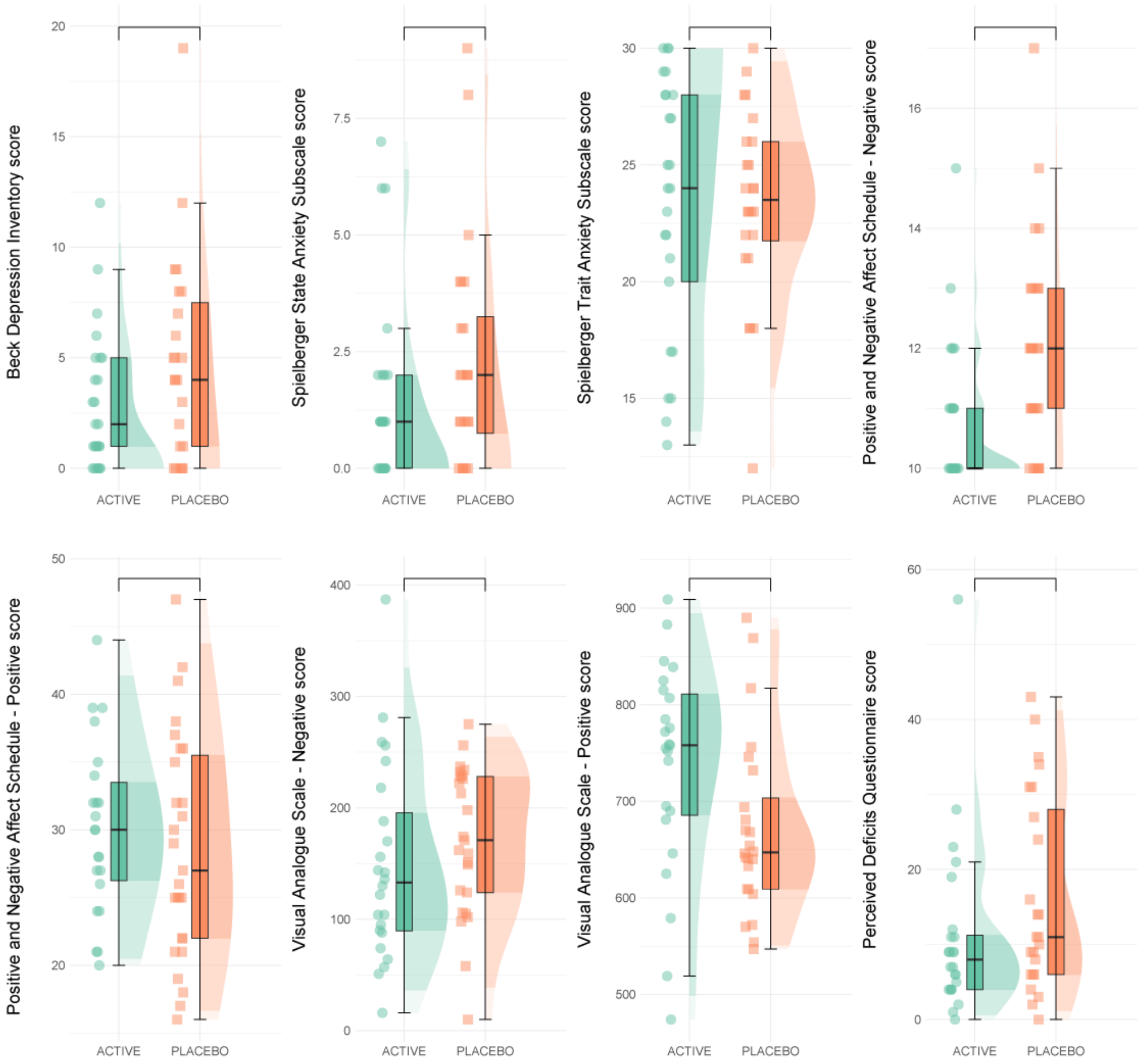

Supplementary Fig. 7. Self-reported Questionnaire measure scores at baseline across allocation groups. At the first study visit (initial dose visit), self-reported participant data was collected across multiple measures. Beck Depression Inventory analysis contained data from *N*=48 individuals. Spielberger subscales (trait/state) contained data from *N*=49 individuals. Positive and Negative Affect Schedule subscales (positive and negative items) contained data from *N*=49 individuals. Visual Analogue Scale subscales (positive and negative items) contained data from *N*=47 individuals. Perceived Deficits Questionnaire analysis contained data from *N*=48 individuals. Boxplots represent the interquartile range (IQR), while the central line depicts the median. The whiskers extend to approximately ± 1.5 times the IQR, encompassing the bulk of the data points; half-violin plots depict the data distribution.

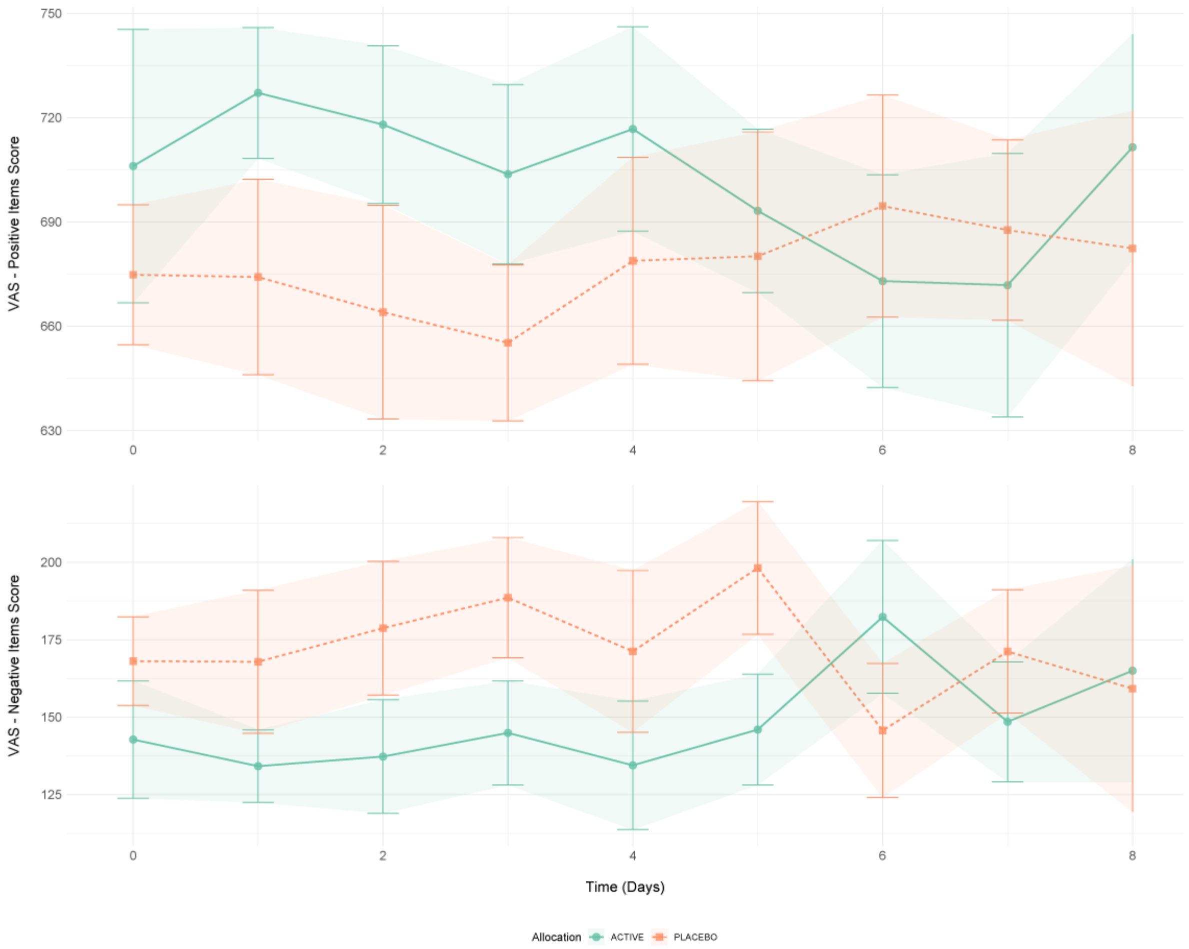

Supplementary Fig. 8. Daily ratings on the Visual Analogue Scale (negative and positive affect items) across allocation groups. At each time point, from day 0 (initial dosing day), participants were asked to make subjective ratings about affect each day. Each subscale contains data from *N*=52 individuals (307 observed ratings for positive subscale ratings; 310 observed ratings for negative subscale ratings; 617 total); lines and plot points depict mean value, with error bars and shaded areas around each line depicting standard mean error.

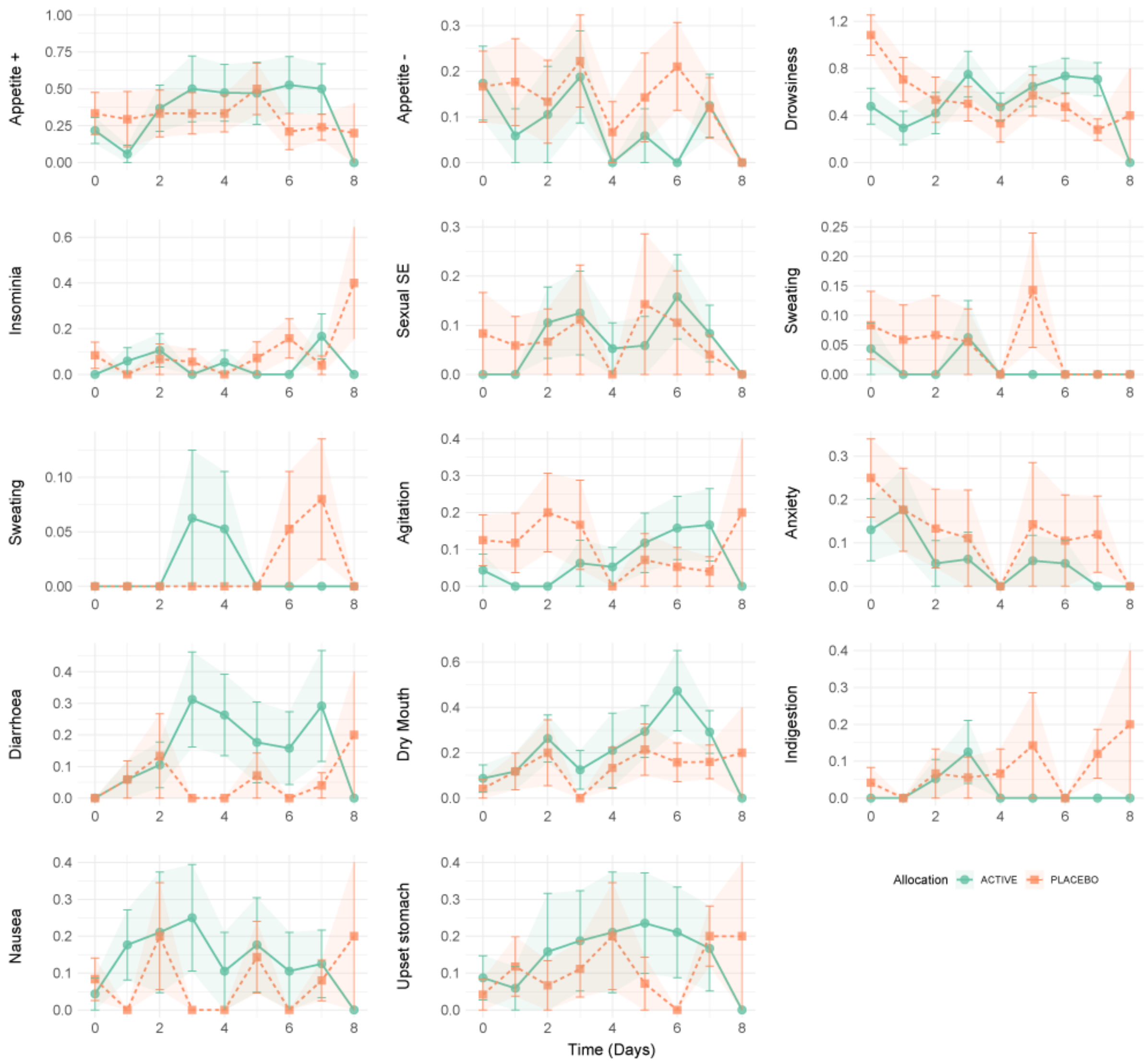

Supplementary Fig. 9. Daily ratings for side effects across allocation groups. At each time point, from day 0 (initial dosing day), participants were asked to rate side effects experienced during the week of drug or placebo administration. Each side effect rating contains data from *N*=52 individuals (309 observed ratings for each side effect item; 4326 total). Side effects items were rated on a scale from 0 – 3 (Not at all – Very Much). Day 0 denotes baseline (initial dosing day); lines and plot points depict mean value, with error bars and shaded areas around each line depicting standard mean error.

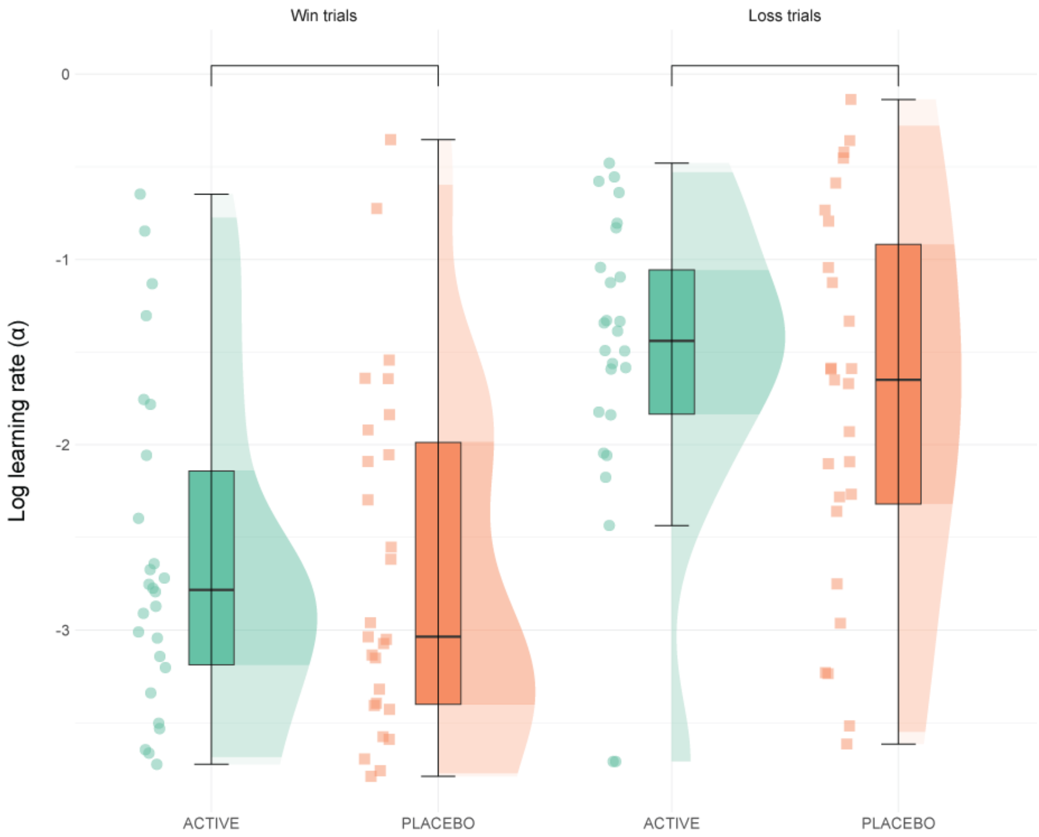

Supplementary Fig. 10. Learning rate (α) parameter during reward and loss learning across allocation groups. The learning rate parameter was fit to participant behaviour across each trial type (win and loss) during the Probabilistic Instrumental Learning Task. All data were derived from models fit to data from *N*=53 individuals; boxplots represent the interquartile range (IQR), while the central line depicts the median. The whiskers extend to approximately ± 1.5 times the IQR, encompassing the bulk of the data points; half-violin plots depict the data distribution.

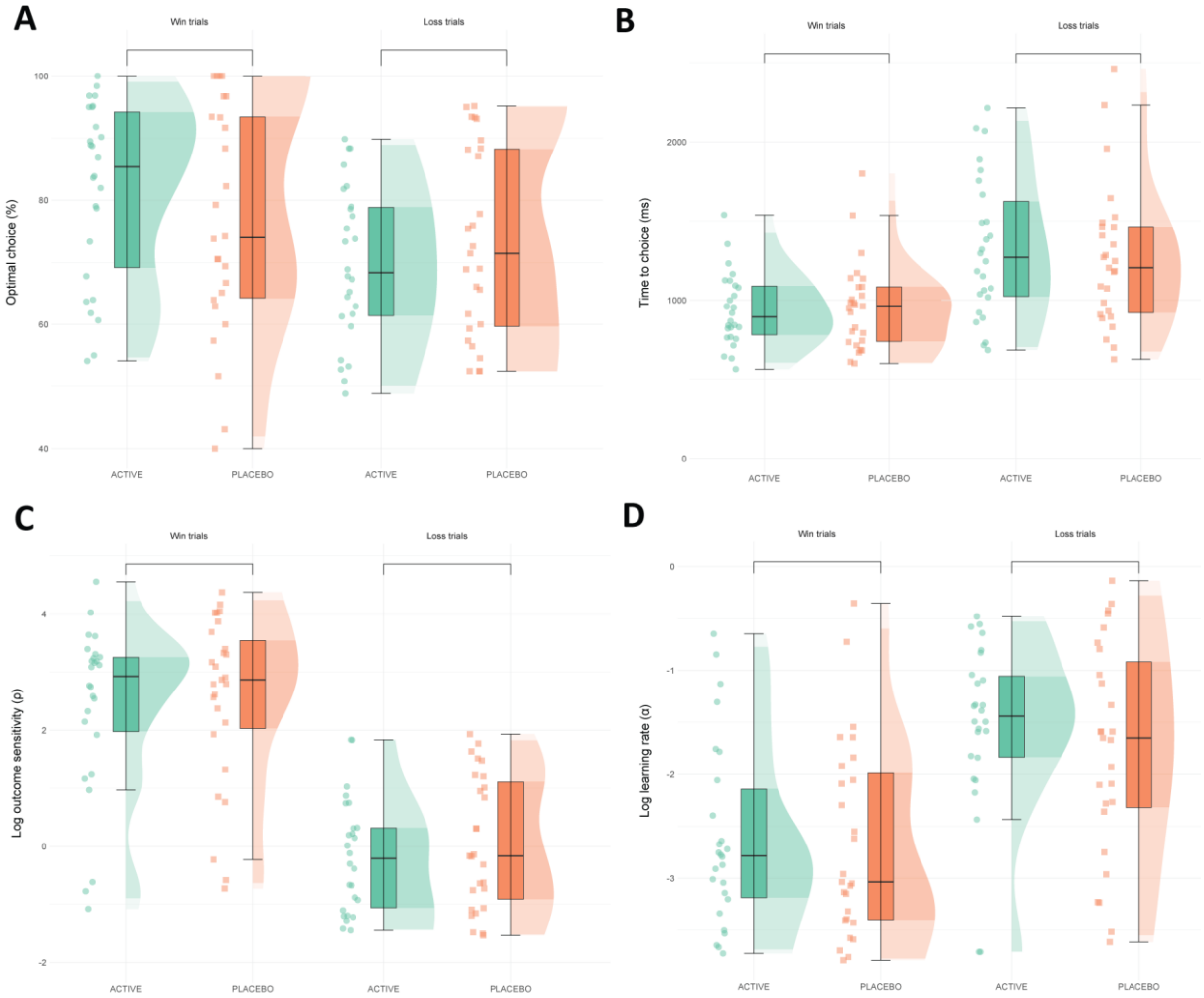

Supplementary Fig. 11. Probabilistic instrumental learning task non-model and model-related outcomes at baseline. A. Optimal choice (%) selection across both trial types. **B.** Time to choice (ms; milliseconds) across both trial types. **C.** Outcome sensitivity (log transformed) across both trial types. **D.** Learning rate (log transformed) across both trial types. Panels **A**-**D**, include data from *N*=53 individuals; boxplots represent the interquartile range (IQR), while the central line depicts the median. The whiskers extend to approximately ± 1.5 times the IQR, encompassing the bulk of the data points; half-violin plots depict the data distribution.

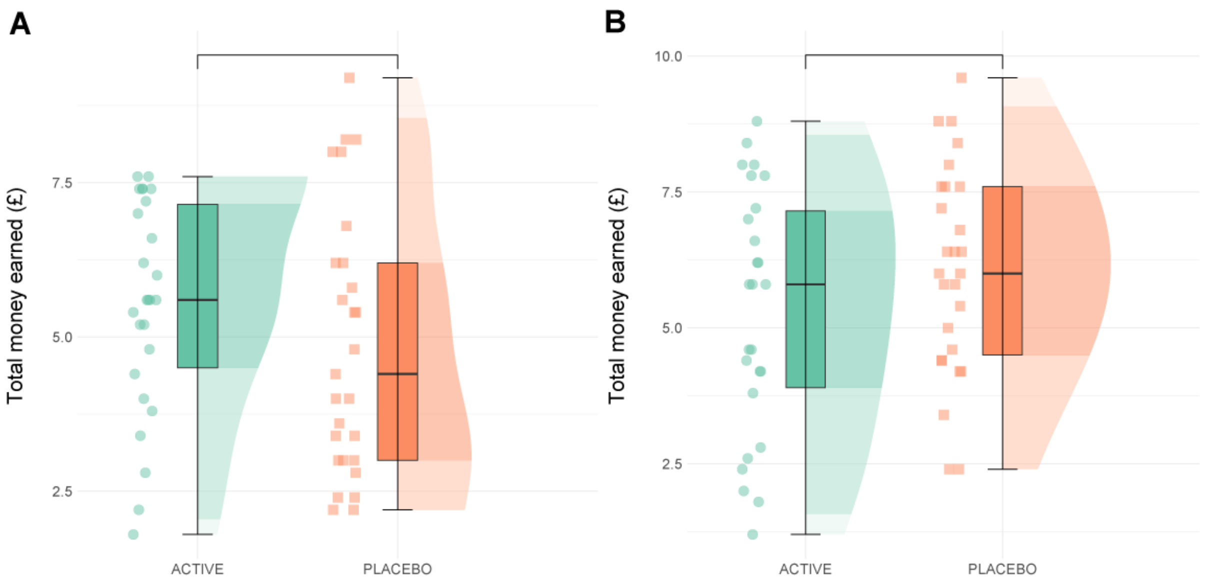

Supplementary Fig. 12. Total money earned on the Probabilistic Instrumental Learning Task – Baseline and Follow-up. A. Total amount earned across allocation groups at baseline in GBP (£). **B.** Total amount of money earned at follow-up across allocation groups in GBP. Panels **A**-**B**, include data from *N*=53 individuals; boxplots represent the interquartile range (IQR), while the central line depicts the median. The whiskers extend to approximately ± 1.5 times the IQR, encompassing the bulk of the data points; half-violin plots depict the data distribution.

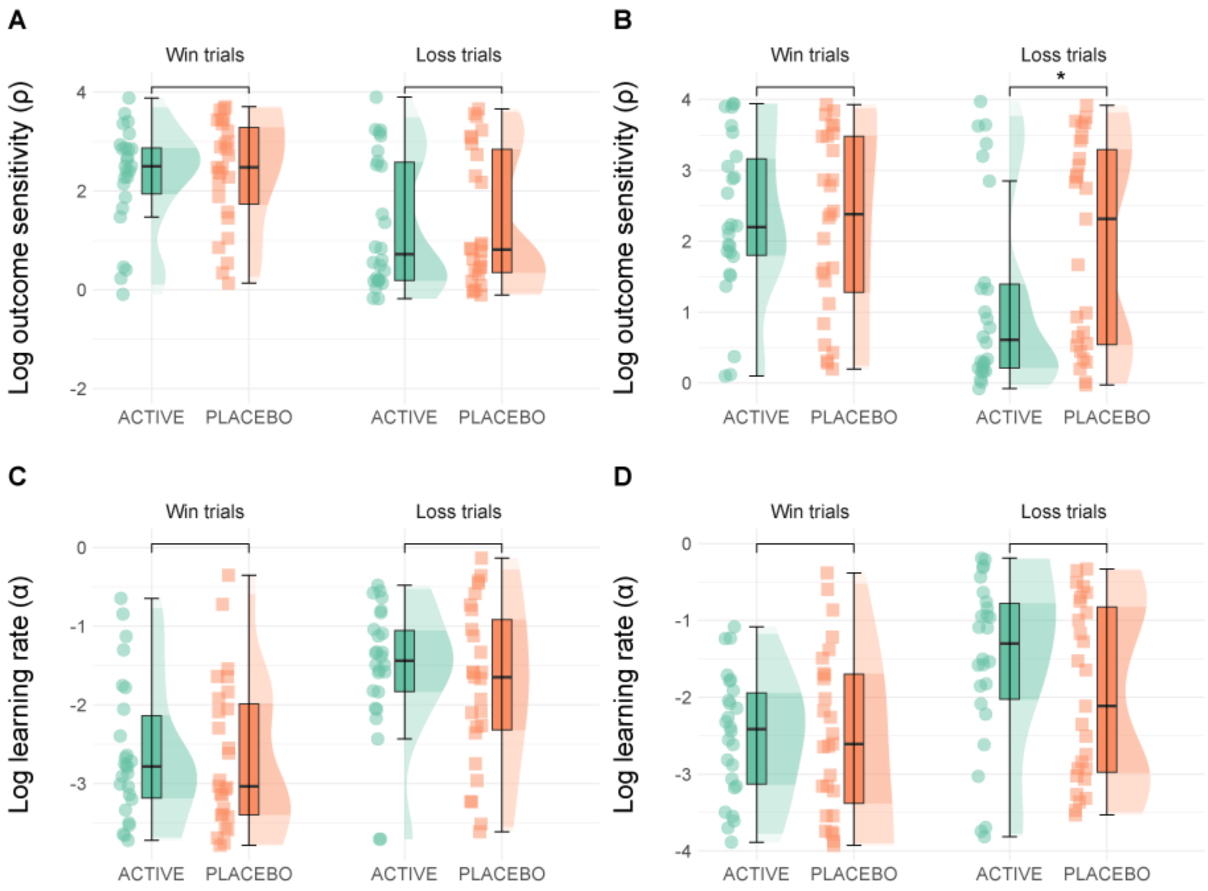

Supplementary Fig. 13. Probabilistic instrumental learning computational modelling – Results LR model without reciprocal updating. A. Outcome sensitivity (ρ) at baseline across allocation groups; B. Reduced outcome sensitivity in the fenfluramine (active) group compared to placebo at follow-up win trials EMM = 0.10 ± 0.36, *p* = 0.78; loss trials EMM = -0.77 ± 0.36, *p* = 0.03). C. and D. Log learning rate (α) at baseline and follow-up, respectively. * *p* ≤ 0.05 indicates group difference by two-tailed EMM tests (Bonferroni-Holm corrected). Panels **A**-**D**, include data from *N*=53 individuals; boxplots represent the interquartile range (IQR), while the central line depicts the median. The whiskers extend to approximately ± 1.5 times the IQR, encompassing the bulk of the data points; half-violin plots depict the data distribution.

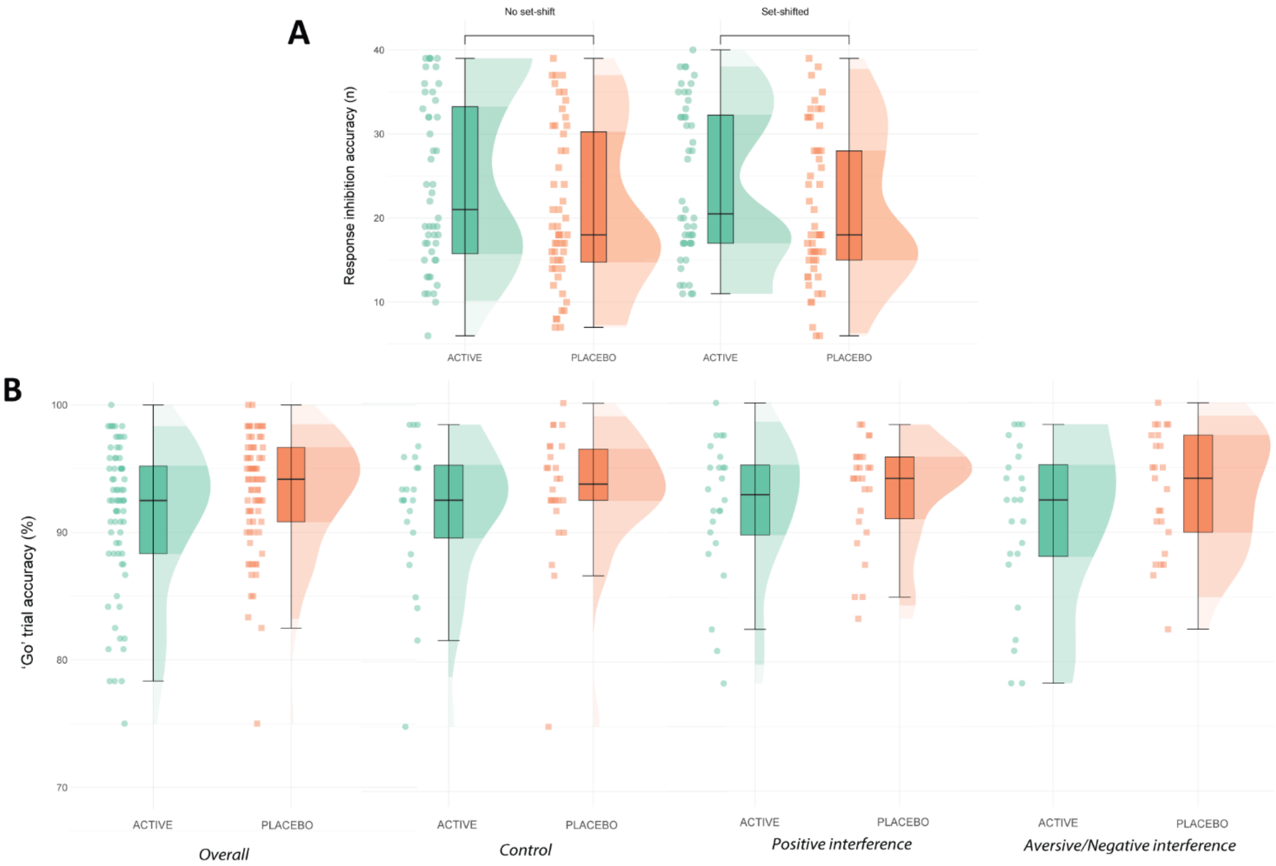

Supplementary Fig. 14. Affective Go/No-Go Task at follow-up: performance across set-shifts and 'go' trial accuracy. A. Response inhibition performance on blocks with set-shifts (rules changing from previous block) or no set-shifts (rule same as previous block) on no-go trial accuracy (response inhibition). **B.** Accuracy for 'go' trial hits across allocation groups. Panels **A**-**B**, include data from *N*=50 individuals; boxplots represent the interquartile range (IQR), while the central line depicts the median. The whiskers extend to approximately ± 1.5 times the IQR, encompassing the bulk of the data points; half-violin plots depict the data distribution.

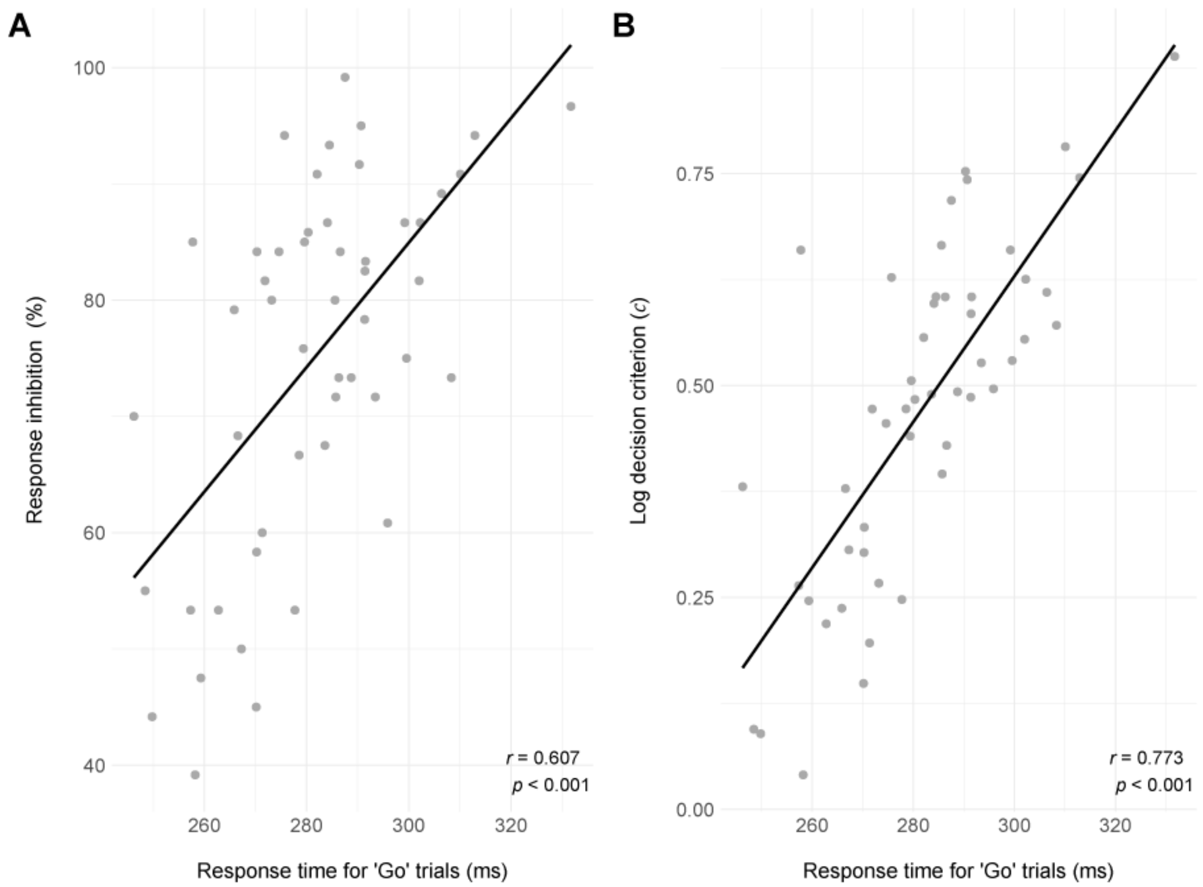

Supplementary Fig. 15. Relationship between 'go' trial response time and both response inhibition and log decision criterion. A. Response times for 'go' trials (ms; milliseconds) increase with response inhibition accuracy (r = 0.61, p = 2.922e-06); B. Response times for 'go' trials (ms; milliseconds) increase with cautious decision-making (log decision criterion [c]) (r = 0.77, p = 4.568e-11). Panel **A**-**B** correlations were produced using two-tailed Pearson's product-moment correlation analysis on task data at follow-up. Panels **A**-**B**, include data from N=50 individuals; line fitted through data points using linear modelling.

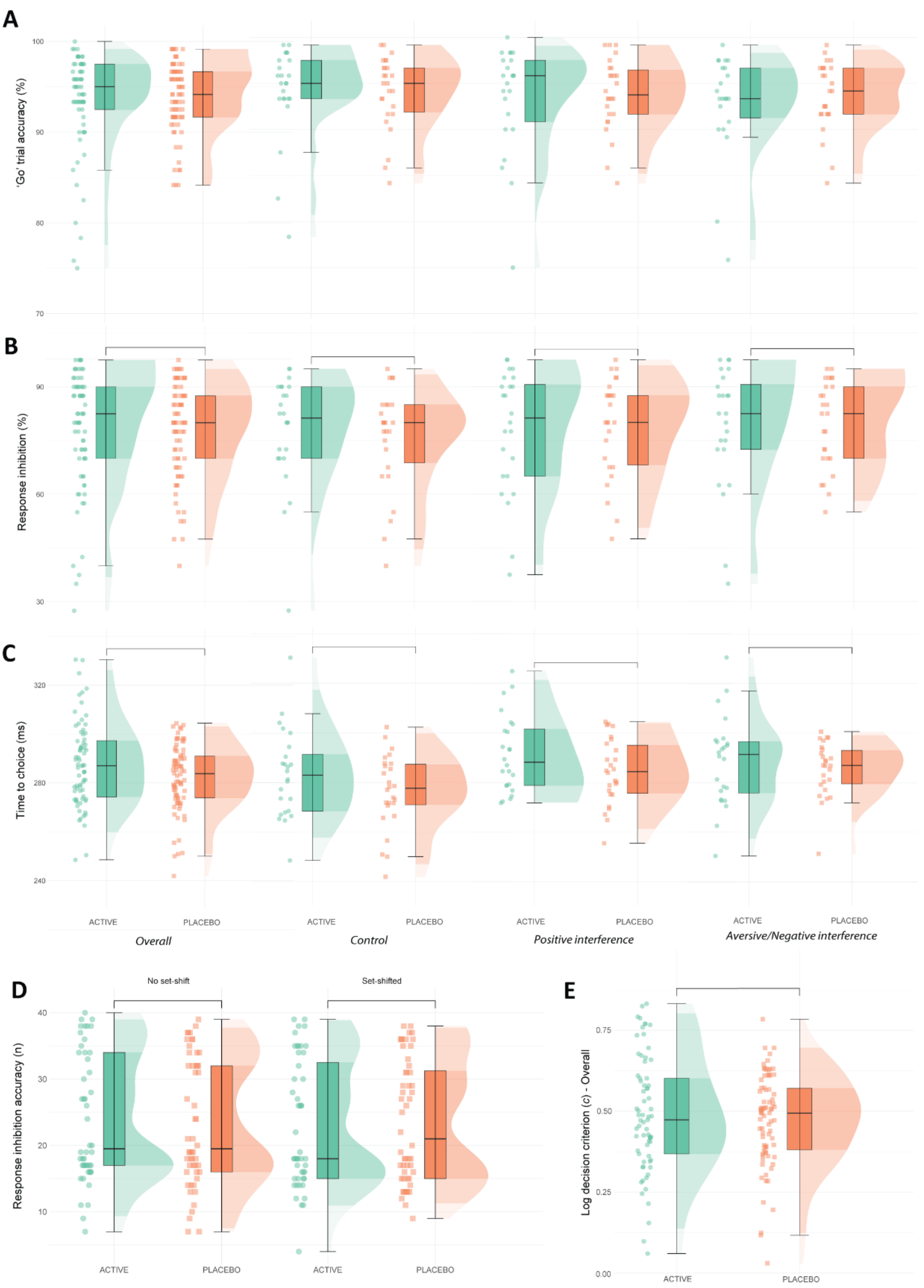

Supplementary Fig. 16. Affective Interference Go/No-Go Task performance at baseline. A. Accuracy for 'go' trials (mean %) across each trial type **B.** Response inhibition (accuracy for 'no-go' trials; mean %) across each trial type. **C.** Time to choice (ms; milliseconds) across each trial type. Panels **A**-**C** share the same columns for trial conditions; left to right: overall, control condition, positive interference and aversive/negative interference. **D.** Response inhibition performance on blocks with set-shifts (rules changing from previous block) or no set-shifts (rule same as previous block) on no-go trial accuracy (response inhibition). E. Signal detection analysis index 'c' decision criterion across each trial type. Panels **A**-**E**, include data from *N*=50 individuals; boxplots represent the interquartile range (IQR), while the central line depicts the median. The whiskers extend to approximately ± 1.5 times the IQR, encompassing the bulk of the data points; half-violin plots depict the data distribution.

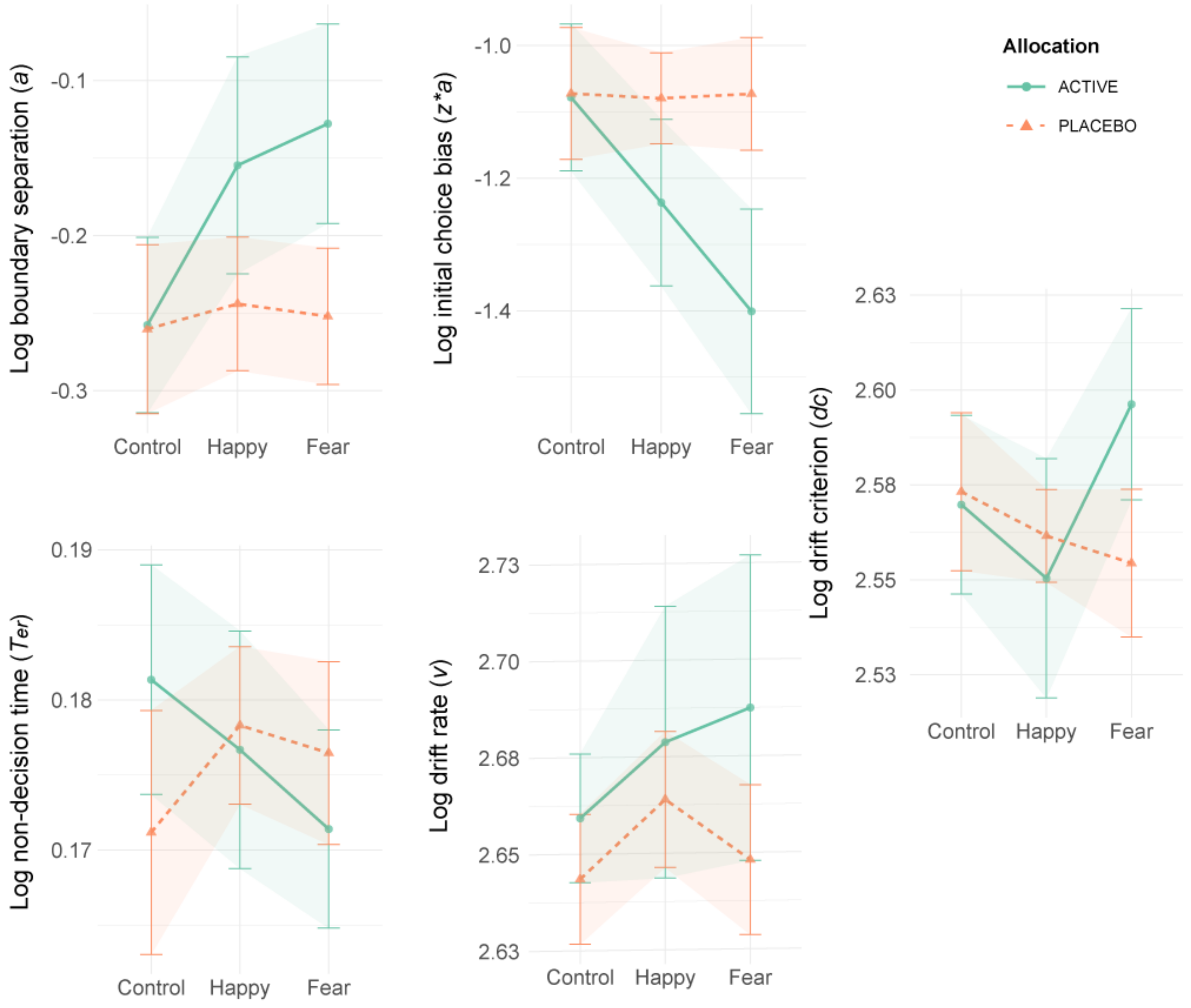

Supplementary Fig. 17. Drift Diffusion Model parameters (Affective Go/No-Go task) across groups and task conditions at follow-up. Drift diffusion model models were fit to data from *N*=50 individuals, producing the following model parameters: 1) Boundary separation (a), which describes the required quantity of evidence for making a decision. 2) Non-decision time (T_{er}) is the period between stimulus onset and the start of the evidence accumulation, where foremost sensory and perceptual processes occur. 3) Initial choice bias (z*a) represents bias toward one of the choice boundaries (a [Go] and 0 [No-go]) at the start of evidence accumulation. 4) Drift rate (v) describes the rate of evidence accumulation before arriving at a choice boundary. 5) Drift criterion (dc) is a constant applied to the mean drift rate which is evidence independent. Lines and plot points depict mean value, with error bars and shaded areas around each line depicting standard mean error.

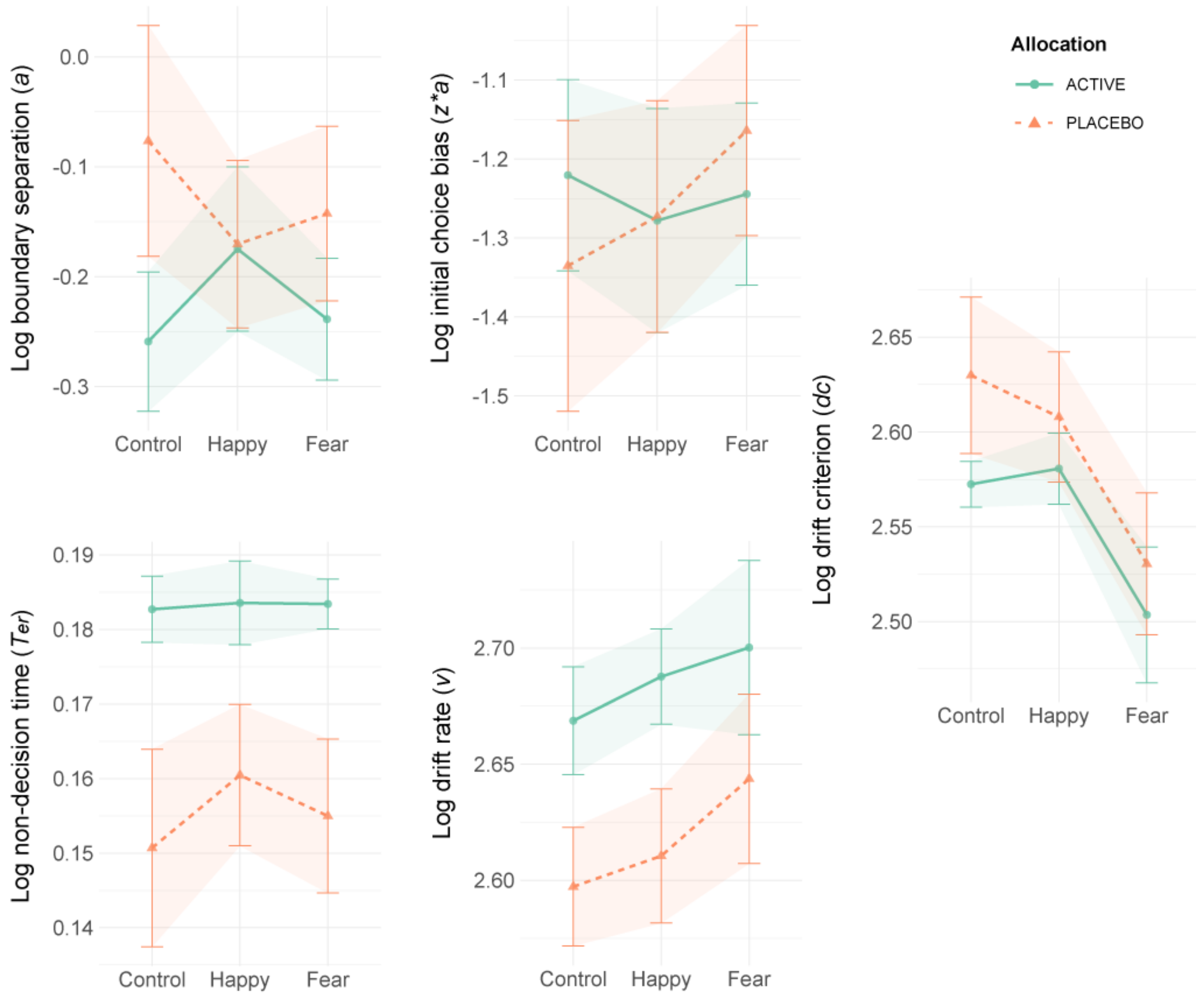

Supplementary Fig. 18. Drift Diffusion Model parameters (Affective Go/No-Go task) across groups and task conditions at baseline. Drift diffusion model models were fit to data from *N*=50 individuals, producing the following model parameters: 1) Boundary separation (a), which describes the required quantity of evidence for making a decision. 2) Non-decision time (T_{er}) is the period between stimulus onset and the start of the evidence accumulation, where foremost sensory and perceptual processes occur. 3) Initial choice bias (z*a) represents bias toward one of the choice boundaries (a [Go] and 0 [No-go]) at the start of evidence accumulation. 4) Drift rate (v) describes the rate of evidence accumulation before arriving at a choice boundary. 5) Drift criterion (dc) is a constant applied to the mean drift rate which is evidence independent. Lines and plot points depict mean value, with error bars and shaded areas around each line depicting standard mean error.

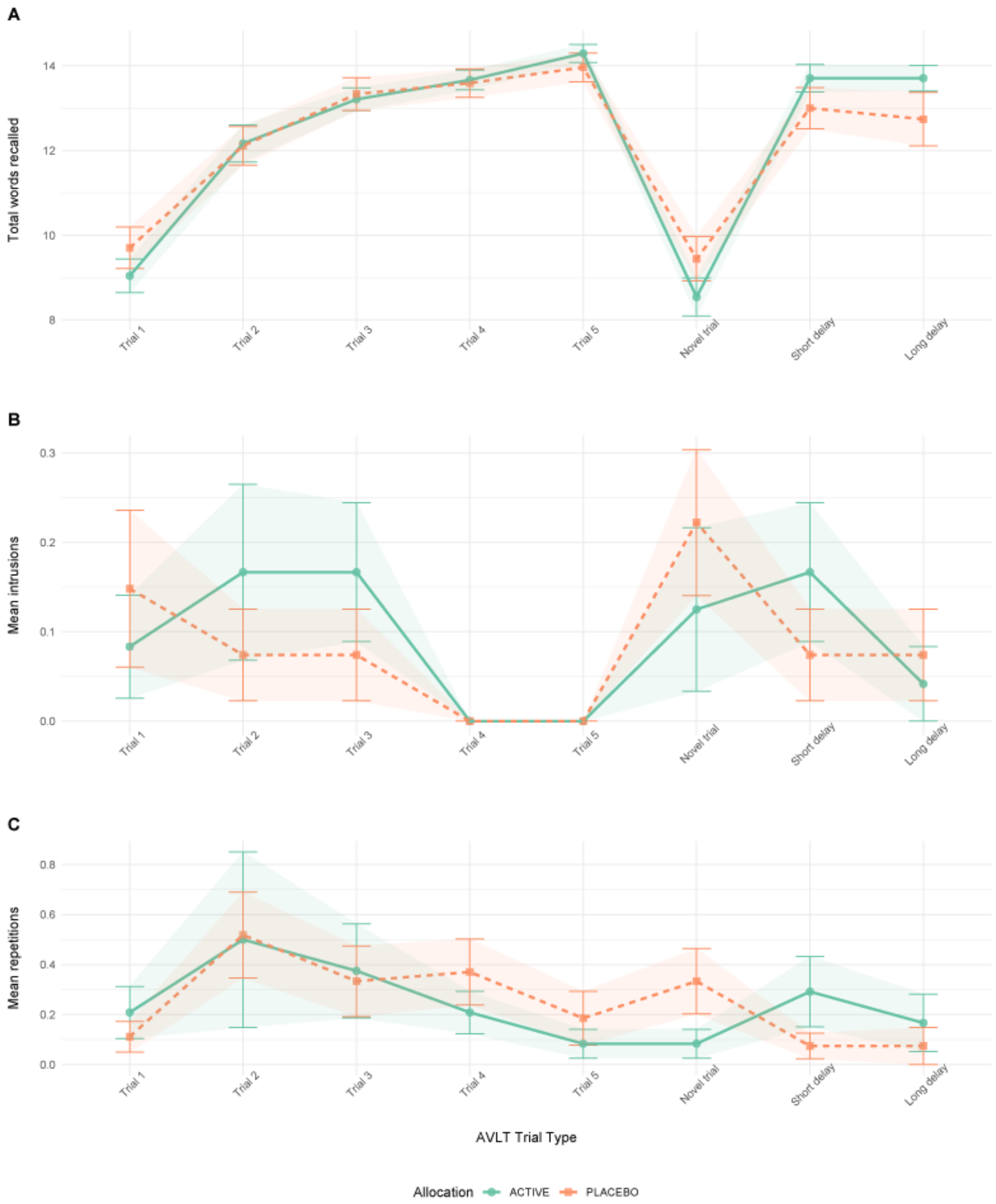

Supplementary Fig. 19. Performance on the Auditory Verbal Learning Task (AVLT) across groups at follow-up. A. Total words recalled across each trial type (Learning [Trials 1 – 5], Novel trial, and Free recall (Short delay and Long delay). **B.** Total number of intrusions (i.e., words falsely recalled) across each trial type. **C.** Total number of repetitions (i.e., true word repetition during recall) across each trial type. All panels contain data from N=51; lines and plot points depict mean value, with error bars and shaded areas around each line depicting standard mean error.

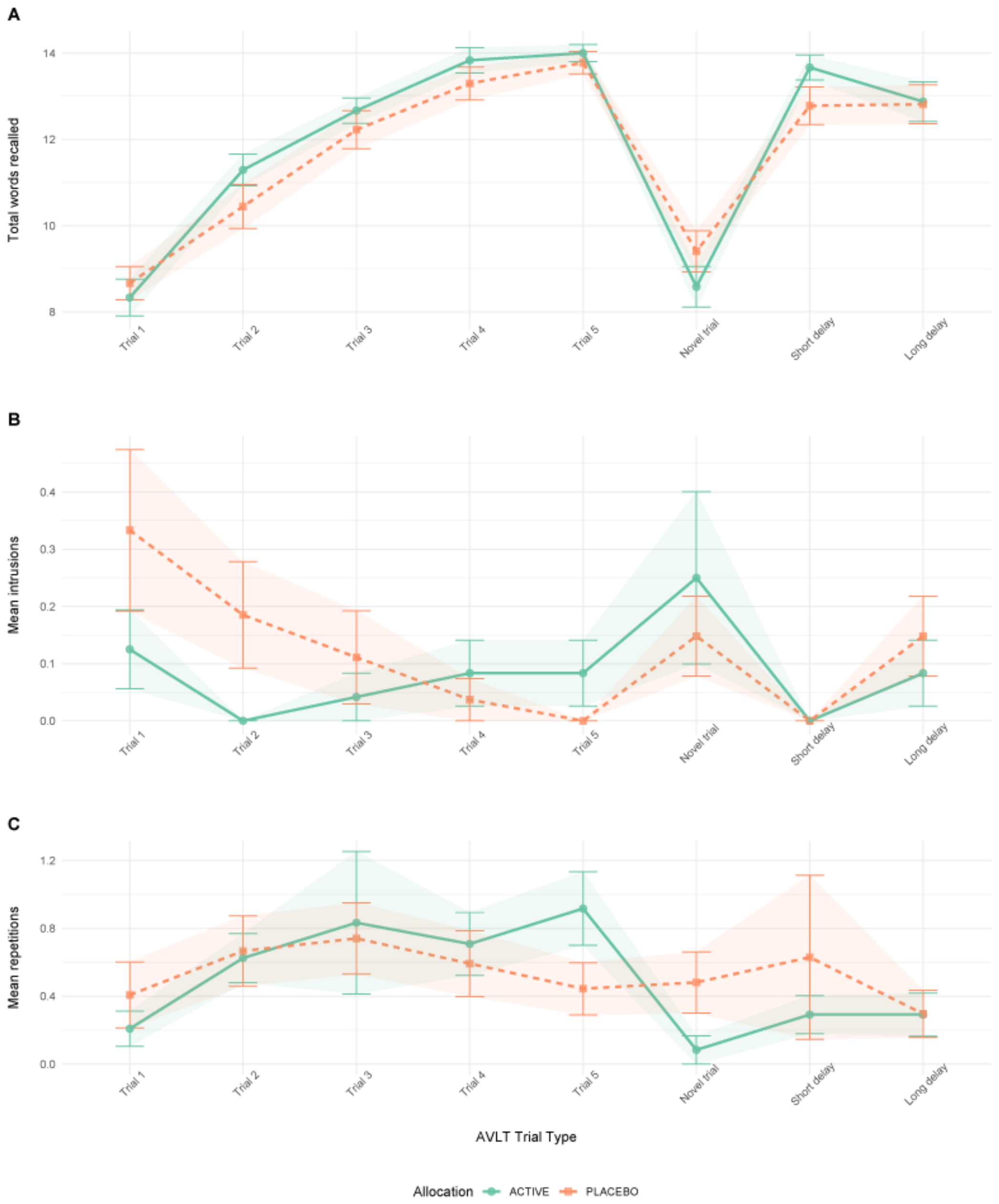

Supplementary Fig. 20. Performance on the Auditory Verbal Learning Task (AVLT) across groups at baseline. A. Total words recalled across each trial type (Learning [Trials 1 – 5], Novel trial, and Free recall (Short delay and Long delay). **B.** Total number of intrusions (i.e., words falsely recalled) across each trial type. **C.** Total number of repetitions (i.e., true word repetition during recall) across each trial type. All panels contain data from N=51; lines and plot points depict mean value, with error bars and shaded areas around each line depicting standard mean error.

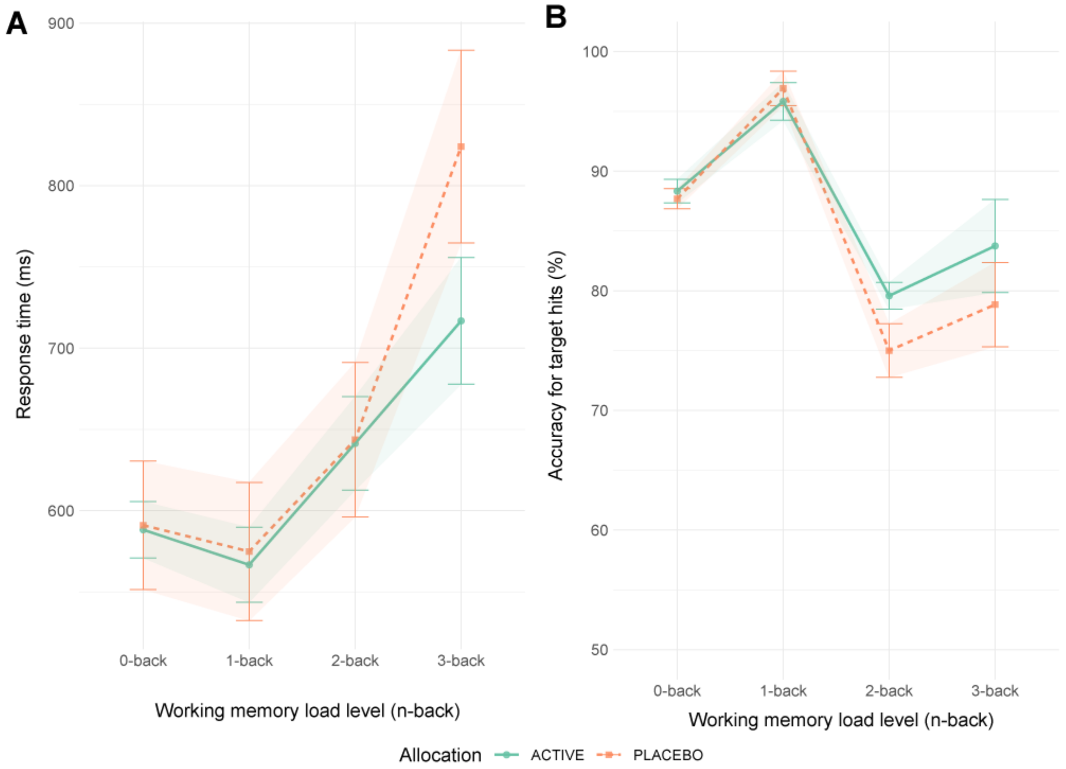

Supplementary Fig. 21. Verbal n-back task performance at baseline. A. Response time (ms; milliseconds) to correct choices across allocation groups. **B.** Accuracy for correct target hits (%) across allocation groups. All panels contain data from N=50; lines and plot points depict mean value, with error bars and shaded areas around each line depicting standard mean error.

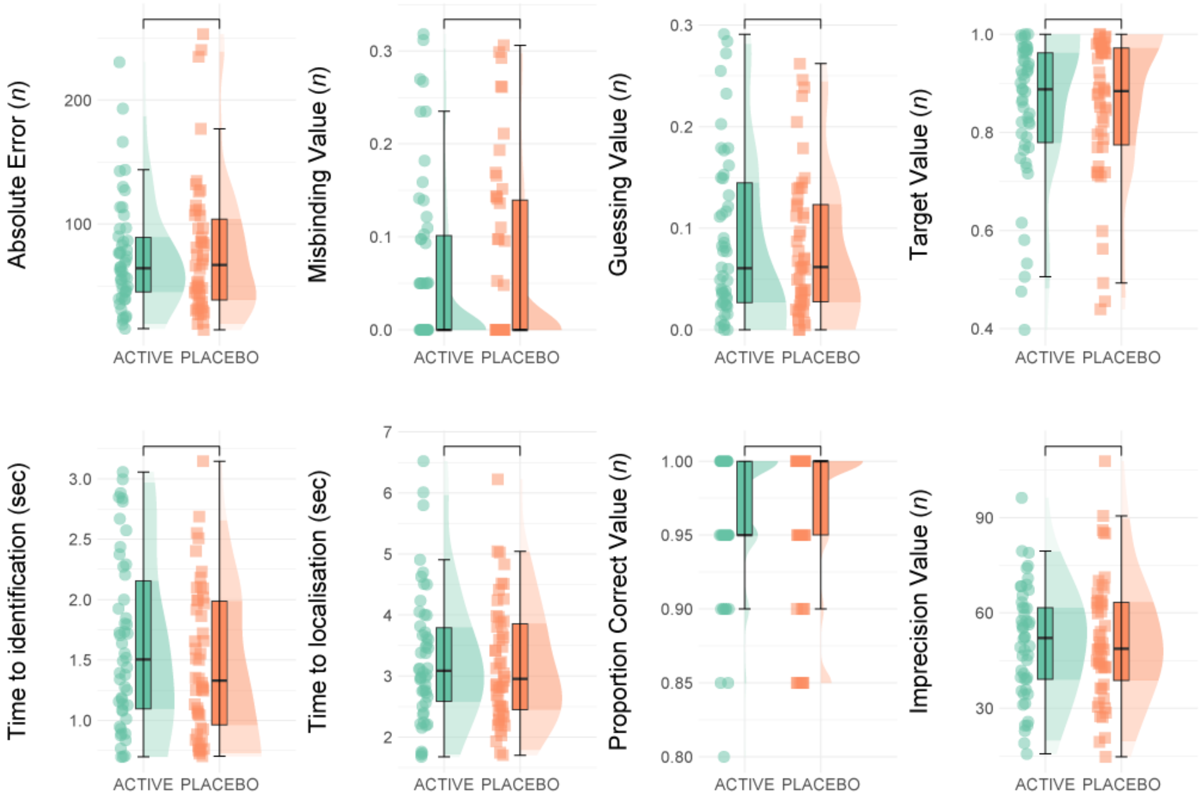

Supplementary Fig. 22. Oxford Memory Test performance at follow-up across allocation groups. The Oxford Memory Test is a test of visuospatial working memory which produces several performance metrics: absolute error, misbinding, guessing values, target values, time to identification, time to localisation, proportion correct value and imprecision value. Each performance metric displayed contains data from *N*=51 individuals; boxplots represent the interquartile range (IQR), while the central line depicts the median. The whiskers extend to approximately ± 1.5 times the IQR, encompassing the bulk of the data points; half-violin plots depict the data distribution.

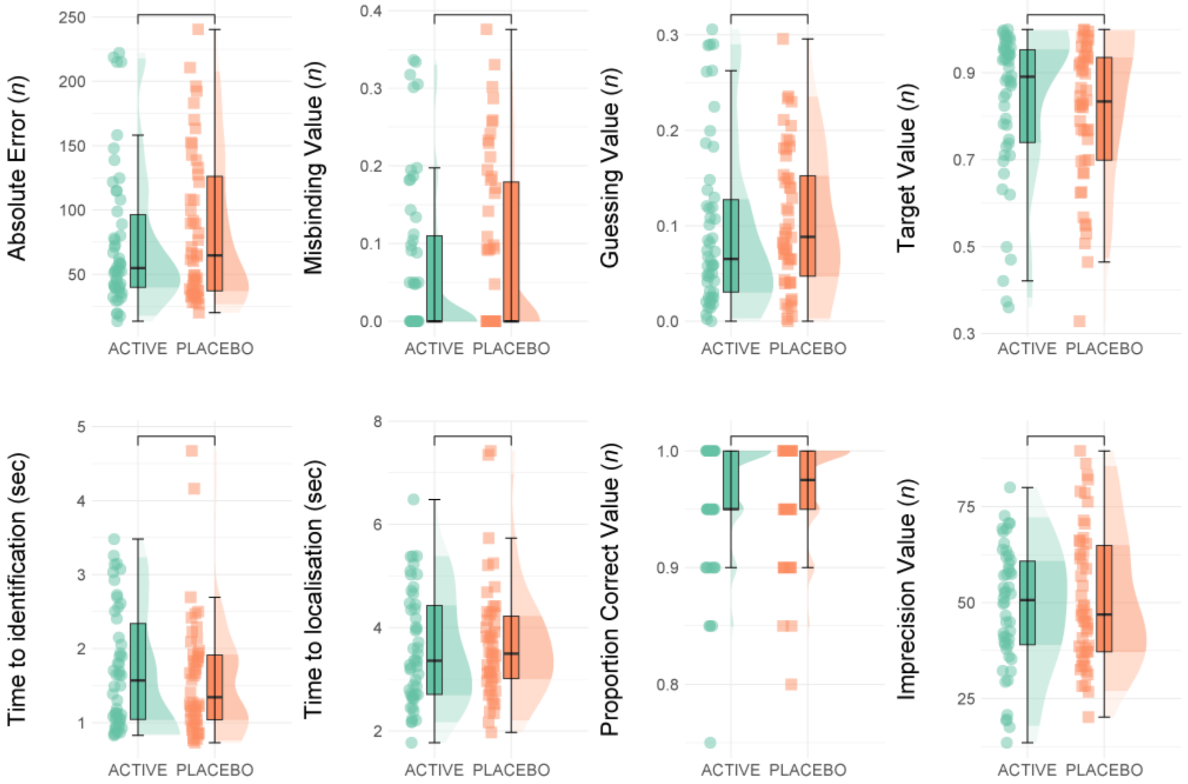

Supplementary Fig. 23. Oxford Memory Test performance at baseline across allocation groups. The Oxford Memory Test is a test of visuospatial working memory which produces several performance metrics: absolute error, misbinding, guessing values, target values, time to identification, time to localisation, proportion correct value and imprecision value. Each performance metric displayed contains data from *N*=51 individuals; boxplots represent the interquartile range (IQR), while the central line depicts the median. The whiskers extend to approximately ± 1.5 times the IQR, encompassing the bulk of the data points; half-violin plots depict the data distribution.

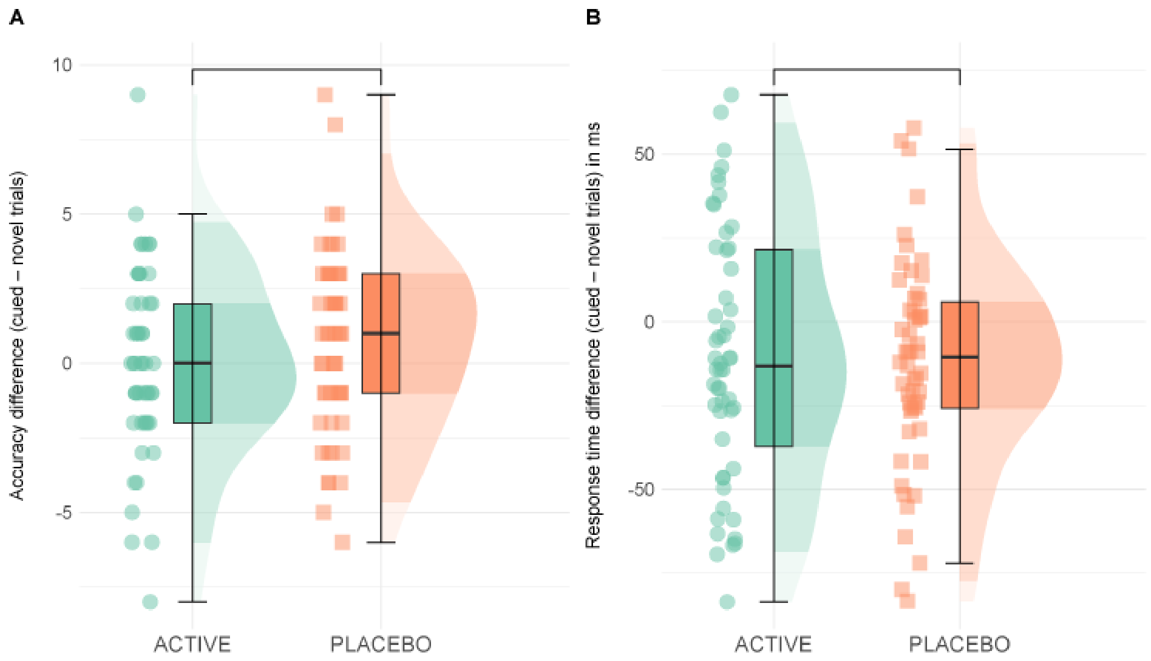

Supplementary Fig. 24. Contextual cueing task performance at follow-up. Panels **A** – **B** accuracy difference (cued – novel trials correct choices) and response time difference (cued – novel trials) (ms; milliseconds), respectively. Panels A – B contain data from *N*=53 individuals; boxplots represent the interquartile range (IQR), while the central line depicts the median. The whiskers extend to approximately ± 1.5 times the IQR, encompassing the bulk of the data points; half-violin plots depict the data distribution.

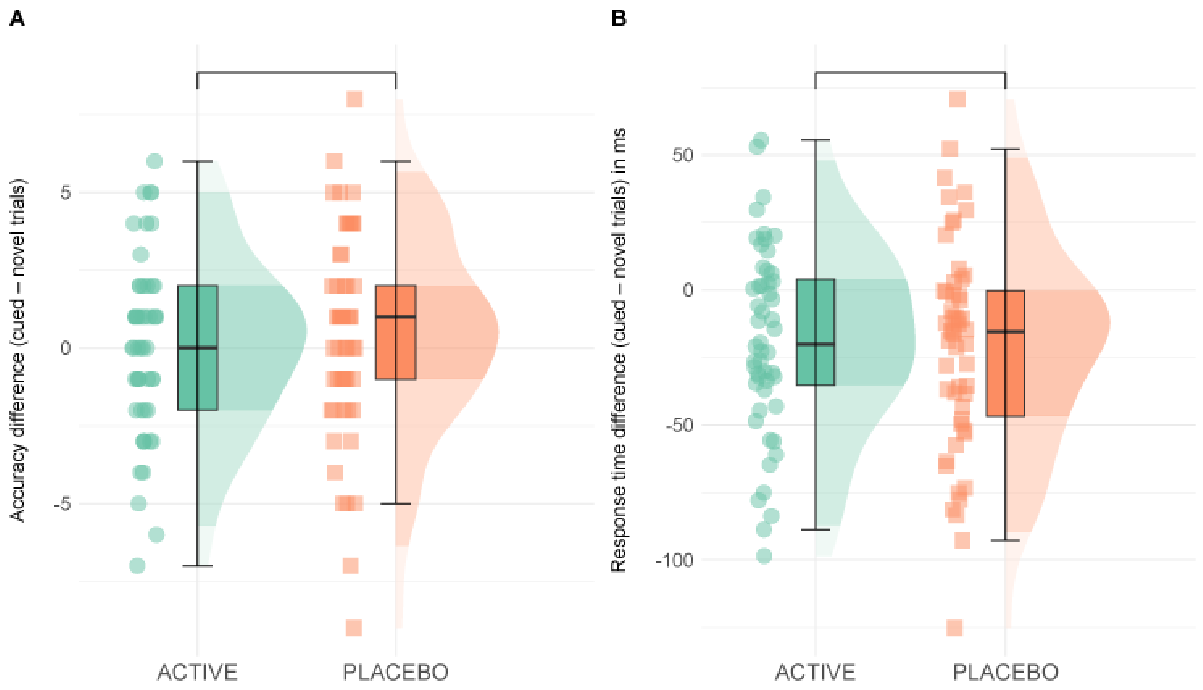

Supplementary Fig. 25. Contextual cueing task performance at baseline. Panels **A – B** accuracy difference (cued – novel trials correct choices) and response time difference (cued – novel trials) (ms; milliseconds), respectively. Panels A – B contain data from *N*=53 individuals; boxplots represent the interquartile range (IQR), while the central line depicts the median. The whiskers extend to approximately ± 1.5 times the IQR, encompassing the bulk of the data points; half-violin plots depict the data distribution.

Supplementary Discussion

Past investigations of the influence of *d-/dl*-fenfluramine on behaviour

Interpretation of our findings in the context of past work on fenfluramine is challenging. The few available neurobehavioral studies of fenfluramine in humans are limited by small and heterogeneous samples, with the most recent published almost two decades ago [24–27]. In this early work, higher doses of *dl*-fenfluramine or d-enantiomer dexfenfluramine were administered which may diminish selectivity for 5-HT [28–31] and have greater potential for neurotoxicity [32–35]. It is important, therefore, that independent attempts to replicate the present findings are undertaken to determine the reliability of low dose fenfluramine as a pro-serotonergic probe.

Our findings align with preliminary work in patients with Dravet Syndrome showed improvements in caregiver ratings of executive functioning following low dose fenfluramine administration [36] .

Consideration of pseudo-specific effects

Research aiming to examine the effect of pharmacological drugs on non-affective cognitive processing must consider the role of pseudo-specificity (*i.e.,* indirect effects of affective processing on cognition [37]), which may be relevant to the SSRA-related enhancement of behavioural inhibition and memory observed in the present findings. However, we observed no statistically significant group differences in potential sources of pseudo-specificity, such as general affective functioning.

Supplementary References

1. Salimetrics, Llc. Expanded range high sensitivity salivary cortisol enzyme immunoassay kit. *Salimetrics, LLC, State College, PA* (2008).

2. Pessiglione, M., Seymour, B., Flandin, G., Dolan, R. J. & Frith, C. D. Dopamine-dependent prediction errors underpin reward-seeking behaviour in humans. *Nature* **442**, 1042–1045 (2006).

3. Gillespie, A. L., Wigg, C., Van Assche, I., Murphy, S. E. & Harmer, C. J. Associations Between Statin Use and Negative Affective Bias During COVID-19: An Observational, Longitudinal UK Study Investigating Depression Vulnerability. *Biol Psychiatry* **92**, 543–551 (2022).

4. Colwell, M. J., Murphy, S. & Harmer, C. J. Emotional Go/No-Go Task (Oxford). (2022) doi:10.5281/ZENODO.6207865.

5. Colwell, M. J., Tagomori, H., Murphy, S. & Harmer, C. N-Back (PERL - Oxford). (2022) doi:10.5281/ZENODO.6208667.

6. Klinge, C. *et al.* Methylphenidate enhances implicit learning in healthy adults. *J Psychopharmacol* **32**, 70–80 (2018).

7. Rey, A. L'examen clinique en psychologie, Paris: Presses Universitaires de France, 1964. *Chemotherapy and objective cognitive functioning* **95**, (1964).

8. Zokaei, N. *et al.* Short-term memory advantage for brief durations in human APOE ε4 carriers. *Sci Rep* **10**, 9503 (2020).

9. Murphy, S., Wright, L., Browning, M., Cowen, P. & Harmer, C. A role for 5-HT4 receptors in human learning and memory. *Psychol Med* (2019) doi:10.1017/S0033291719002836.

10. Halahakoon, D. C. *et al.* Pramipexole Enhances Reward Learning by Preserving Value Estimates. *Biol Psychiatry* (2023) doi:https://doi.org/10.1016/j.biopsych.2023.05.023.

11. Browning, M., Paulus, M. & Huys, Q. J. M. What is computational psychiatry good for? *Biol Psychiatry* **93**, 658–660 (2023).

12. McHugh, S. B. *et al.* Aversive prediction error signals in the amygdala. *Journal of Neuroscience* **34**, 9024–9033 (2014).

13. Sengupta, A. & Holmes, A. A Discrete Dorsal Raphe to Basal Amygdala 5-HT Circuit Calibrates Aversive Memory. *Neuron* **103**, 489-505.e7 (2019).

14. Makowski, D. The psycho package: An efficient and publishing-oriented workflow for psychological science. *J Open Source Softw* **3**, 470 (2018).

15. Pallier, C. Computing discriminability and bias with the R software. Preprint at (2002).

16. de Gee, J. W. *et al.* Pupil-linked phasic arousal predicts a reduction of choice bias across species and decision domains. *Elife* **9**, e54014 (2020).

17. Ratcliff, R., Huang-Pollock, C. & McKoon, G. Modeling individual differences in the go/no-go task with a diffusion model. *Decision* **5**, 42 (2018).

18. Patil, A., Huard, D. & Fonnesbeck, C. J. PyMC: Bayesian stochastic modelling in Python. *J Stat Softw* **35**, 1 (2010).

19. Wiecki, T. V, Sofer, I. & Frank, M. J. HDDM: Hierarchical Bayesian estimation of the drift-diffusion model in Python. *Front Neuroinform* 14 (2013).

20. Nguyen, K. P., Josić, K. & Kilpatrick, Z. P. Optimizing sequential decisions in the drift–diffusion model. *J Math Psychol* **88**, 32–47 (2019).

21. Miłkowski, M., Hensel, W. M. & Hohol, M. Replicability or reproducibility? On the replication crisis in computational neuroscience and sharing only relevant detail. *J Comput Neurosci* **45**, 163–172 (2018).

22. Forster, M. R. Key concepts in model selection: Performance and generalizability. *J Math Psychol* **44**, 205–231 (2000).

23. Gamerman, D. & Lopes, H. F. *Markov Chain Monte Carlo: Stochastic Simulation for Bayesian Inference*. (CRC press, 2006).

24. Caycedo, N., Connell, J., Hellewell, J. S. E. & Deakin, J. F. W. Effects of fenfluramine on executive and other cognitive functions: 6.3. *Behavioural Pharmacology* **6**, 33 (1995).

25. Lichtenberg, P. *et al.* Effect of fenfluramine on mood: A double-blind placebo-controlled trial. *Biol Psychiatry* **31**, 351–356 (1992).

26. Bond, A. J., Feizollah, S. & Lader, M. H. The effects of d-fenfluramine on mood and performance, and on neuroendocrine indicators of 5-HT function. *Journal of Psychopharmacology* **9**, 1–8 (1995).

27. Luciana, M., Collins, P. F. & Depue, R. A. Opposing roles for dopamine and serotonin in the modulation of human spatial working memory functions. *Cereb Cortex* **8**, 218 (1998).

28. Storlien, L. H. & Smythe, G. A. d-Fenfluramine effects on hypothalamic monoamine activities and their hormonal correlates. *Brain Res* **597**, 60–65 (1992).

29. Zaczek, R. *et al.* Effects of repeated fenfluramine administration on indices of monoamine function in rat brain: pharmacokinetic, dose response, regional specificity and time course data. *Journal of Pharmacology and Experimental Therapeutics* **253**, 104–112 (1990).

30. Baumann, M. H., Ayestas, M. A. & Rothman, R. B. In vivo correlates of central serotonin function after high-dose fenfluramine administration. *Ann N Y Acad Sci* **844**, 138–152 (1998).

31. Molliver, D. C. & Molliver, M. E. Anatomic evidence for a neurotoxic effect of (±)-fenfluramine upon serotonergic projections in the rat. *Brain Res* **511**, 165–168 (1990).

32. Baumann, M. H., Ayestas, M. A., Dersch, C. M. & Rothman, R. B. 1-(m-Chlorophenyl)piperazine (mCPP) Dissociates In Vivo Serotonin Release from Long-Term Serotonin Depletion in Rat Brain. *Neuropsychopharmacology* **24**, 492–501 (2001).

33. Erenburg, N., Hamed, R., Shaul, C., Perucca, E. & Bialer, M. Comparative activity of the enantiomers of fenfluramine and norfenfluramine in rodent seizure models, and relationship with their concentrations in plasma and brain. *Epilepsia* (2023).

34. Baumann, M. H., Ayestas, M. A. & Rothman, R. B. Functional consequences of central serotonin depletion produced by repeated fenfluramine administration in rats. *Journal of Neuroscience* **18**, 9069–9077 (1998).

35. Kalia, M. Reversible, short-lasting, and dose-dependent effect of (+)-fenfluramine on neocortical serotonergic axons. *Brain Res* **548**, 111–125 (1991).

36. Bishop, K. I. *et al.* Improved everyday executive functioning following profound reduction in seizure frequency with fenfluramine: Analysis from a phase 3 long-term extension study in children/young adults with Dravet syndrome. *Epilepsy & Behavior* **121**, 108024 (2021).

37. Colwell, M. J. *et al.* Pharmacological targeting of cognitive impairment in depression: recent developments and challenges in human clinical research. *Transl Psychiatry* **12**, 484 (2022).
