# Supplementary material for: Direct serotonin release in humans shapes aversive learning and inhibition
Source: Nat Commun. 2024 Aug 9;15:6617. doi: 10.1038/s41467-024-50394-x (PMC11315928; doi:10.1038/s41467-024-50394-x)
Supplement: Supplementary file 3 — Reporting Summary [file 41467_2024_50394_MOESM3_ESM.pdf]

nature portfolio

Corresponding author(s): Michael Colwell; Catherine Harmer

Last updated by author(s): 06/02/2024

Reporting Summary

Nature Portfolio wishes to improve the reproducibility of the work that we publish. This form provides structure for consistency and transparency in reporting. For further information on Nature Portfolio policies, see our Editorial Policies and the Editorial Policy Checklist.

Statistics

For all statistical analyses, confirm that the following items are present in the figure legend, table legend, main text, or Methods section.

n/a	Confirmed	
☐	☒	The exact sample size (*n*) for each experimental group/condition, given as a discrete number and unit of measurement
☐	☒	A statement on whether measurements were taken from distinct samples or whether the same sample was measured repeatedly
☐	☒	The statistical test(s) used AND whether they are one- or two-sided<br>*Only common tests should be described solely by name; describe more complex techniques in the Methods section.*
☐	☒	A description of all covariates tested
☐	☒	A description of any assumptions or corrections, such as tests of normality and adjustment for multiple comparisons
☐	☒	A full description of the statistical parameters including central tendency (e.g. means) or other basic estimates (e.g. regression coefficient) AND variation (e.g. standard deviation) or associated estimates of uncertainty (e.g. confidence intervals)
☐	☒	For null hypothesis testing, the test statistic (e.g. F, t, r) with confidence intervals, effect sizes, degrees of freedom and P value noted<br>*Give P values as exact values whenever suitable.*
☒	☐	For Bayesian analysis, information on the choice of priors and Markov chain Monte Carlo settings
☒	☐	For hierarchical and complex designs, identification of the appropriate level for tests and full reporting of outcomes
☐	☒	Estimates of effect sizes (e.g. Cohen's *d*, Pearson's *r*), indicating how they were calculated

Our web collection on statistics for biologists contains articles on many of the points above.

Software and code

Policy information about availability of computer code

Data collection	MATLAB 2021a; Psychtoolbox 3; PsychoPy 2021.1.4; Anaconda 2021.4; Presentation (Neurobs) 23.0; Qualtrics Surveys
Data analysis	R (4.3.1); MATLAB (R2022a); Python (version 3.8.8); Docker 4.22.0. The following R packages were utilised: dplyr (1.1.2), tidyverse (2.0.0), gtools (3.9.4), knitr (1.42), data.table (1.14.8), ggplot2 (3.4.2), car (3.1-2), ggbeeswarm (0.7.2), ggrepel (0.9.3), readxl (1.4.2), openxlsx (4.2.5.2), ggpubr (0.6.0), rstatix (0.7.2), "ez" (4.4-0), ggsignif (0.6.4), RColorBrewer (1.1-3), emmeans (1.8.5), plotrix (3.8-2), sdamr (0.2.0), cowplot – (1.1.1), psycho (0.6.1), ggridges (0.5.4), viridis (0.6.4), ggstance (0.3.6), ggdist (3.3.0), gghalves (0.1.4), ggpp (0.5.4), lme4 (1.1-33), stringr (1.5.0), effectsize (0.8.6), lmerTest (3.1-3), TOSTER (0.8.1). All required Python dependencies are included within the Docker image: hcp4715/hddm:0.8. No additional MATLAB packages required.<br><br>All preprocessing and analysis code are available on GitHub: https://github.com/mjcolwell/SSRA_human_behaviour_data_and_code.

For manuscripts utilizing custom algorithms or software that are central to the research but not yet described in published literature, software must be made available to editors and reviewers. We strongly encourage code deposition in a community repository (e.g. GitHub). See the Nature Portfolio guidelines for submitting code & software for further information.

Data

Policy information about availability of data

All manuscripts must include a data availability statement. This statement should provide the following information, where applicable:

- Accession codes, unique identifiers, or web links for publicly available datasets
- A description of any restrictions on data availability
- For clinical datasets or third party data, please ensure that the statement adheres to our policy

Full code and availability statements are included within the manuscript. All analysis scripts and data are available on GitHub: https://github.com/mjcolwell/SSRA_human_behaviour_data_and_code.

Research involving human participants, their data, or biological material

Policy information about studies with human participants or human data. See also policy information about sex, gender (identity/presentation), and sexual orientation and race, ethnicity and racism.

Reporting on sex and gender	Research participants were asked to self-report their gender, which was initially used to characterise the sample. The potential covariance of gender with treatment effects across study outcomes was investigated post-hoc via ANCOVA modeling. These analyses are reported in full in the Supplementary Results.
Reporting on race, ethnicity, or other socially relevant groupings	Research participants were asked about family history of mental health difficulties, educational background and first spoken language. These factors were used to characterise the sample, but did not inform the primary analyses.
Population characteristics	The final sample consisted of N=53 individuals (mean age = 20.15; 32 females). For the Probabilistic Instrumental Learning and Contextual Cueing tasks, the data consisted of N=53 individuals (mean age = 20.15; 32 females). For the Auditory Verbal Learning Task, the data consisted of N=51 individuals (mean age = 20.15; 31 females). For the Oxford Memory Task, the data consisted of N=51 individuals (mean age = 20.16; 31 females). For the Affective Interference Go/No-Go Task, the data consisted of N=50 individuals (mean age = 20.22; 32 females). For the Verbal n-back task, the data consisted of N=50 individuals (mean age = 20.1; 31 females). This sample is representative of the local recruitment area, Oxford, which has a substantial population of younger students.
Recruitment	Participants were recruited using online (Meta advertising; CallForParticipants) and local (leaflets) advertisements. Using this recruitment strategy, we did not anticipate self-selection bias within the study. Prior to study participation, participants provided informed consent. Participants were reimbursed 175 GBP upon completion of their participation in the research.
Ethics oversight	The study was approved by the University of Oxford Central University Research Ethics Committee (reference R69642/RE005).

Note that full information on the approval of the study protocol must also be provided in the manuscript.

Field-specific reporting

Please select the one below that is the best fit for your research. If you are not sure, read the appropriate sections before making your selection.

☐ Life sciences ☒ Behavioural & social sciences ☐ Ecological, evolutionary & environmental sciences

For a reference copy of the document with all sections, see nature.com/documents/nr-reporting-summary-flat.pdf

Behavioural & social sciences study design

All studies must disclose on these points even when the disclosure is negative.

Study description	Quantitative research study involving a drug intervention in healthy adults without mental/physical health difficulties and not using a psychoactive medication.
Research sample	Fifty-six individuals were randomised to the drug or placebo. Three drop-outs/exclusions occurred post-randomisation due to low adherence, adverse effects or failing to meet the inclusion criteria. The final sample was 53 participants. Participant flow is described in detail within the CONSORT diagram (Fig 6) in the Methods section. Statistical tests were undertaken to ensure homogeneity of demographic variables between allocation groups. All participants underwent full medical screening (including EEG, blood work and psychiatric history) prior to participation. A sample of young healthy adults was selected as this closely matched clinical population for which fenfluramine is licensed (young persons with Dravet Epilepsy), allowing safer investigation of the drug.
Sampling strategy	The sampling strategy was random sampling. Participants were randomised using a stratified block algorithm. Sample size power calculations were undertaken to produce a target sample size for between-analysis at the follow-up visit (N=52). Statistical power was increased by using baseline performance data as a covariate within ANCOVA models.

Data collection	Experimental task data was collected in a laboratory setting using computer and pen-and-paper. Computer task data was collected using a 120hz monitor, using Psychopy, MATLAB/Psychtoolbox and Presentation (Neurobs) software packages. Questionnaire data was collected using Qualtrics software. Salivary cortisol data was collected using self-administered Salivette cotton swabs. Blood pressure data was collected using a OMRON blood pressure device. Drug or placebo adherence was assessed by collecting the remaining IMP (investigational medicinal product) and checking against the expected remaining dosage. Researchers and participants were not aware of allocation (double-blind design).
Timing	Data collection lasted from June 2021 until June 2022.
Data exclusions	Data collected from participants which dropped out/were excluded post-randomisation was excluded from the final analysis (n=3). For some experimental tasks within the battery (e.g. AVLT, OMT or Go/No-Go), participant data was excluded due to misunderstanding task rules or missing task data (n=1-3).
Non-participation	Of the ninty-five individuals screened for study participation, thirty-five did not meet the inclusion criteria and four declined invitation to participate. Fifty-six individuals who met the full inclusion criteria were randomised to the drug or placebo. Three drop-outs post-randomisation occurred due to exclusion criteria (n=1), adverse events (n=1), or self-reported low adherence (n=1).
Randomization	Randomisation occured externally (Clinical Pharmacy Support Unit, Oxford Health NHS Foundation Trust) using a stratified block randomisation algorithm.

Reporting for specific materials, systems and methods

We require information from authors about some types of materials, experimental systems and methods used in many studies. Here, indicate whether each material, system or method listed is relevant to your study. If you are not sure if a list item applies to your research, read the appropriate section before selecting a response.

Materials & experimental systems

n/a	Involved in the study
☒	☐ Antibodies
☒	☐ Eukaryotic cell lines
☒	☐ Palaeontology and archaeology
☒	☐ Animals and other organisms
☐	☒ Clinical data
☒	☐ Dual use research of concern
☒	☐ Plants

Methods

n/a	Involved in the study
☒	☐ ChIP-seq
☒	☐ Flow cytometry
☒	☐ MRI-based neuroimaging

Clinical data

Policy information about clinical studies

All manuscripts should comply with the ICMJE guidelines for publication of clinical research and a completed CONSORT checklist must be included with all submissions.

Clinical trial registration	NCT05026398
Study protocol	The version-controlled protocol document first uploaded in 20th January 2021 and associated changes can be accessed here: https://osf.io/p3gc4/?view_only=137cf73b1c974357a096f5d0df84757a.
Data collection	Data collection occurred within the Neurosciences Building, Warneford Hospital (Oxford). This was a research laboratory setting. Recruitment began in April 2020 and ended completed in June 2021. Data collection began in June 2020 and ended in June 2021.
Outcomes	Primary and secondary outcomes were defined on the clinical trials registration, and are defined as changes in the drug group (vs placebo) at follow-up:<br><br>• Primary outcome 1: Change in Go/No-Go Task performance (measured by Accuracy on the Go/No-Go task).<br>• Primary outcome 2: Change in Auditory Verbal Learning Task (measured by Accuracy on AVLT).<br>• Primary outcome 3: Change in N-back task performance (measured by accuracy on the N-back task).<br><br>• Secondary outcome 1: Changes in reward sensitivity (measured by reward sensitivity on the Probabilistic Instrumental Learning Task<br>• Secondary outcome 2: Changes in categorisation of emotional words (measured by accuracy to categorise positive and negative descriptor words).<br>• Secondary outcome 3: Changes in recall of emotional words (measured by number of words accurately recalled).<br>• Secondary outcome 4: Changes in recognition of emotional facial expressions (measured by accuracy of emotion labels (e.g. disgusted face) assigned by participants to expressive faces which have appeared on a computer screen for a period of 500ms).<br>• Secondary outcome 5: Changes in visual short term memory on the Oxford Memory Test (OMT) (measured by Accuracy on the Oxford Memory Task).<br>• Secondary outcome 6: Changes in visual search ability (measured by accuracy during contextual cueing task (CCT)).<br>• Secondary outcome 7: Changes in control measures of subjective state (measured by ratings on the Positive and Negative Affect

Schedule).

This was an experimental medicine study conducted with healthy individuals, and therefore is not technically classified as a clinical trial.

Plants

Seed stocks	NA
Novel plant genotypes	NA
Authentication	NA
